# Beyond Dealumination: Does Fluorine Reshape Zeolitic Acidity?

**DOI:** 10.1021/jacs.6c04557

**Published:** 2026-06-11

**Authors:** Lu Song, Natalia Morlanés, Edy Abou−Hamad, Javier Ruiz-Martínez

**Affiliations:** † 127355 King Abdullah University of Science and Technology, KAUST Catalysis Center (KCC), Thuwal 23955, Saudi Arabia; ‡ 127355 King Abdullah University of Science and Technology, Core lab, solid-state NMR, Thuwal 23955, Saudi Arabia

## Abstract

Fluoride-containing media have been widely adopted in the post-treatment of ZSM-5 zeolites for heterogeneous catalysis. Their effects have predominantly been discussed in terms of the well-established dealumination process. However, comparatively little attention has been paid to the influence of residual fluorine on the local chemical environment, which governs zeolitic acidity and catalytic performance. In this work, mild fluorination of ZSM-5 was achieved by controlling the ammonium fluoride (NH_4_F) content during hydrothermal treatment. Our findings reveal that fluorine induces significant perturbations in the acidic properties of ZSM-5, distinct from the conventional dealumination effect. To elucidate these effects at the atomic level, we employed ^27^Al, ^19^F, ^27^Si, and ^1^H solid-state magic angle spinning (MAS) nuclear magnetic resonance (NMR) spectroscopy, which provided detailed chemical and structural insights. The correlated incorporation of fluorine within the zeolite framework leads to strong local polarization, thereby enabling the protons to exhibit Brønsted acid site (BAS)-like behavior. Comparative analysis with nonaluminum-containing silicalite-1 further confirmed the fluorine-induced acidity, as supported by two-dimensional (2D) ^1^H double-quantum single-quantum (DQ–SQ) MAS NMR results. Catalytic testing in the methanol-to-hydrocarbon (MTH) reaction demonstrated that such fluorinated ZSM-5 exhibits an extended catalytic lifetime along with enhanced aromatic selectivity, underscoring the pivotal electronic influence of fluorine beyond its classic role in dealumination.

## Graphical abstract

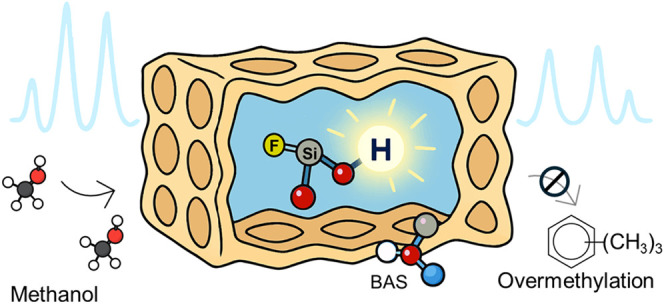

## Introduction

Zeolites have long been recognized as archetypal solid acids, serving as indispensable catalysts in heterogeneous catalysis.^1,2^ The catalytic performance primarily arises from the presence of Brønsted acid sites (BASs), where protons are bound to bridging hydroxyl oxygens within the zeolite framework (Si–OH–Al).^3^ These protons act as active centers that mediate molecular adsorption and proton transfer, initiating the formation and transformation of reactive intermediates in acid-catalyzed reactions.^4^ However, excessive acidity or high BAS density often accelerates undesired secondary reactions such as excessive hydrogen transfer and polyaromatic coke formation, in methanol-to-hydrocarbon reactions (MTH), for instance, leading to rapid deactivation.^5,6^

Driven by these challenges, extensive efforts have been devoted to modifying the acidity to achieve optimal catalytic performance. Accordingly, diverse strategies have been developed to tailor the acid properties of zeolites through compositional^7^ or postsynthetic modifications.^5,8^ Conventional approaches primarily modulate zeolitic acidity by altering the T-site framework environment with heteroatoms, where the BAS originates. The acid strength primarily depends on the polarization of the bridging hydroxyl group, which determines the ease of proton release.^9^ This polarization, in turn, is controlled by the electronegativity of the T atom, following the order Al­(OH)­Si > Ga­(OH)Si > Fe­(OH)Si > In­(OH)Si > B­(OH)­Si.^10,11^ Substitution of Al by heteroatoms has therefore been widely explored to moderate zeolitic acidity. For instance, Yaripour et al.^12^ reported that the isomorphous substitution of B into H-ZSM-5 significantly enhanced catalyst lifetime in the methanol-to-olefins reaction by reducing the ratio of strong to mild acid sites and suppressing coke formation.

Beyond framework Al substitution, postsynthetic dealumination is typically achieved through steaming or chemical etching, which can effectively reduce the BAS density and, in some cases, enhance molecular diffusion.^13,14^ Steaming, although widely applied,^15^ is notoriously difficult to control and frequently leads to the formation of abundant extra-framework Al (EFAl) clusters,^15^ thereby complicating the correlation between the framework structure and acidity. In this regard, fluoride-assisted chemical etching using hydrofluoric acid (HF)^16^ or ammonium fluoride (NH_4_F)^17^ has emerged as a distinct postsynthetic route. Unlike steaming, fluoride-mediated modification proceeds through a different chemical pathway, involving the selective extraction of framework and extra-framework Al under controlled conditions.^18^ In this context, fluorine, owing to its exceptionally high electronegativity (3.98),^19^ provides a powerful handle to modify the local electronic field of the zeolite framework beyond its conventional role in dealumination. Early studies, as summarized by Ghosh et.al., demonstrated that fluorination can enhance the acidity and catalytic performance of oxide catalysts, an effect attributed to the strong electronegativity of fluorine.^20^ This established an early conceptual link between fluorination and acidity enhancement. However, an atomic-level understanding of how fluorine alters the framework’s electronic environment and the nature of resulting acid properties remains limited, motivating the need to elucidate the intrinsic role of fluorine beyond dealumination.

Hence, in this work, we systematically investigated the structural and electronic consequences induced by fluorine in ZSM-5 zeolites. The fluorinated ZSM-5 samples were prepared under mild fluorination conditions using aqueous NH_4_F, with the NH_4_F content optimized below 600 mg g_zeolite_^–1^ to minimize excessive framework dealumination. Acidity characterization using NH_3_-TPD and pyridine-adsorbed Fourier transform infrared (FTIR) showed that F-ZSM-5 exhibited enhanced acidity despite the partial framework Al extraction. To establish a direct link between fluorine incorporation and changes in acidic properties, a combination of FTIR and solid-state nuclear magnetic resonance (NMR) spectroscopy was employed to elucidate the fluoride-induced structural and electronic modifications in the zeolite framework. In contrast to aluminum-free Silicalite-1, the presence of framework aluminum enables strong perturbations arising from the high electronegativity of fluorine. This approach provides atomic-level insights into the intrinsic role of fluorine beyond the well-stablished effects of dealumination.

## Experiments

### Catalyst Synthesis

ZSM-5 (ammonium type) zeolite (Zeolyst, CBV 8014) was used for the parent catalyst. For the fluorination of ZSM-5, 50, 75, 100, 150, 200, 300, 600, 1800, and 2400 mg of ammonium Fluoride (NH_4_F) were dissolved in 20 mL of deionized water, respectively. 1.0 g of parent catalyst was hydrothermally treated with the above-mentioned fluorine-containing aqueous solutions at 140 °C for 10 h. The resulting products were washed with deionized water 5 times and then dried at 60 °C overnight. Lastly, parent ZSM-5 and the dried catalysts were calcined at 500 °C for 5 h, with a ramping rate of 1 °C min^–1^, and the resulting samples were denoted as Fx-ZSM-5, where *x* represents the NH_4_F loading normalized to the zeolite amount for easy comparison, defined as the multiple of 100 mg NH_4_F per gram of zeolite. Additionally, two selectively prepared sample through method-2 (denoted as M2) were prepared by mixing ZSM-5 with 35 or 75 mg of NH_4_F in DI water (10 mL), directly drying at 90 °C for 12 h without hydrothermal treatment and DI-water washing, and subsequently calcining under the same condition as mentioned above, the resulted samples were denoted as M2-F0.35 and M2-F0.75, respectively.

Detailed experimental procedures, characterization methods, and catalytic testing conditions are provided in the Supporting Information.

## Results and Discussion

Although ammonium fluoride (NH_4_F) has been widely employed for the post-treatment of zeolites due to its controllable and mild fluorination behavior compared with hydrofluoric acid (HF).^21^ Excessive NH_4_F can still result in severe dealumination and structural collapse because of the formation of soluble Al–F complexes.^14,22^ In addition, fluoride treatment can also induce changes in morphology and crystal accessibility, which may affect coke formation behavior.^23^ To ensure structural integrity while enabling fluorine incorporation, NH_4_F was employed under carefully optimized conditions to prepare F-ZSM-5 samples, as schematically illustrated in Figure 1a. The amount of NH_4_F dissolved in deionized (DI) water for the hydrothermal treatment of ZSM-5 ranged from 100 to 2400 mg per gram of zeolite (mg g_zeolite_^–1^), and the resulting samples were denoted as Fx-ZSM-5 (where *x* = 1–24). The optimized NH_4_F content was determined to be below 600 mg g_zeolite_^–1^, which effectively avoided extensive dealumination as confirmed by the inductively coupled plasma-optical emission spectroscopy (ICP-OES) results shown in Figure 1b. Specifically, the Si/Al ratio increased from 40 in the parent ZSM-5 to 46 in F1-ZSM-5, 47 in F3-ZSM-5 and 50 in F6-ZSM-5, indicating a limited degree of dealumination under mild fluorination. In contrast, a significant loss of Al occurred at the highest NH_4_F content (2400 mg g_zeolite_^–1^), where the Si/Al ratio sharply increased to 60, indicating extensive dealumination (∼32% loss of Al). This trend is further corroborated by X-ray fluorescence (XRF) analysis (Figure S1), which shows a progressive increase in the Si/Al ratio with increasing NH_4_F content. Accordingly, the fluorination conditions were deliberately restricted to a controlled NH_4_F range (NH_4_F ≤ 600 mg g_zeolite_^–1^) to minimize the confounding effects arising from severe dealumination and potential framework collapse, such as defect formation and loss of microporosity.^24^

**Figure 1:**
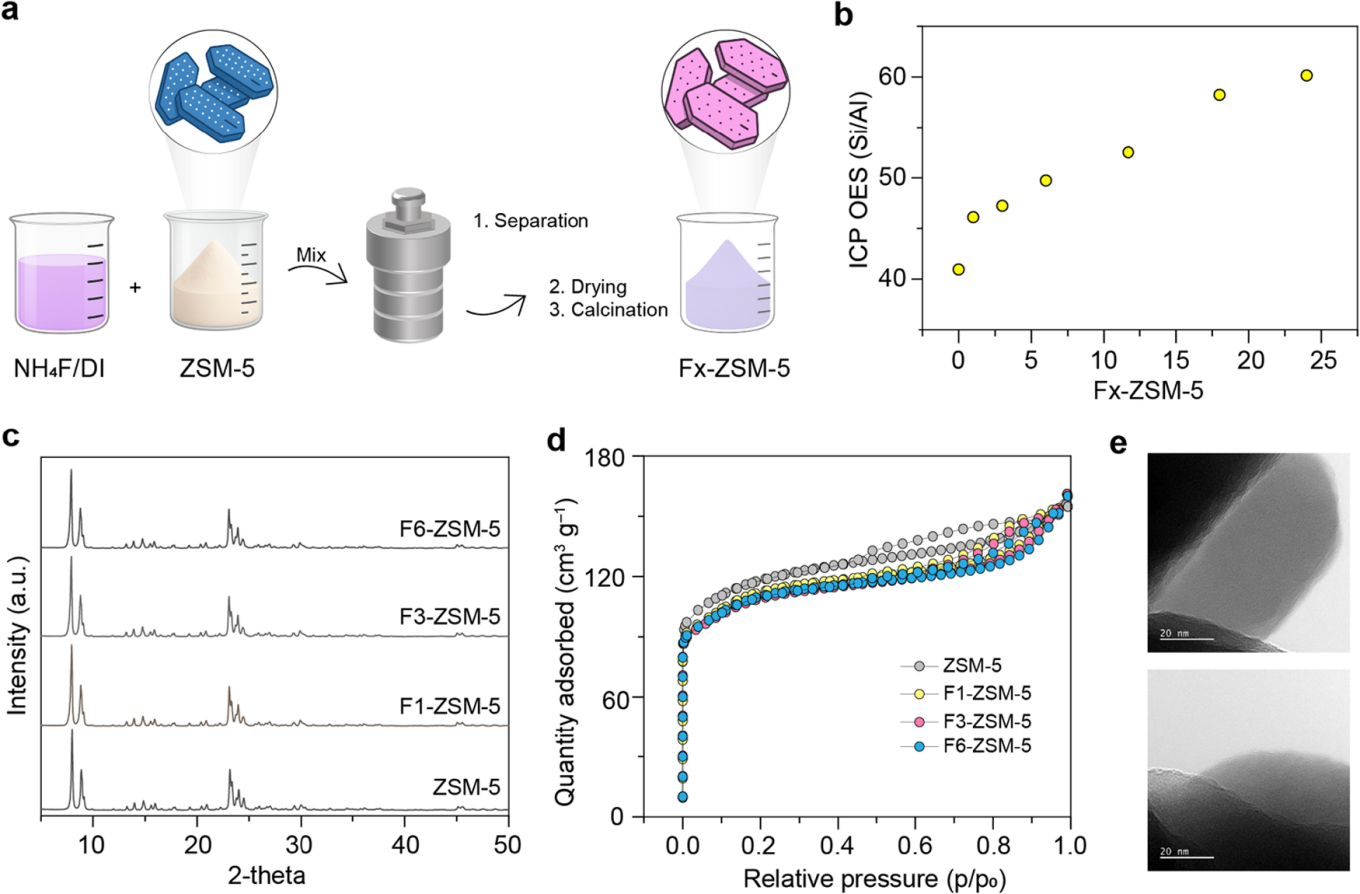
(a) Schematic illustration of the synthesis of the F-ZSM-5 samples. (b) ICP-OES results of the Si/Al ratios of modified ZSM-5 with increasing F content. (c) PXRD patterns and (d) N_2_ physisorption isotherms of parent ZSM-5 and F-ZSM-5 catalysts. (e) HRTEM images of parent ZSM-5 (top) and F3-ZSM-5 (bottom).

To verify that the crystal structure of the fluorinated ZSM-5 remained intact within the controlled NH_4_F range, powder X-ray diffraction (PXRD) analysis was performed on the fluorinated samples. Maryam *et*. *al*.^24^ previously reported that harsh fluorination conditions used for dealumination purposes can lead to a pronounced loss of crystallinity in ZSM-5. In contrast, the optimized mild fluorination treatment (NH_4_F ≤ 600 mg g_zeolite_^–1^) applied in this work effectively preserved the framework, as evidenced by the retention of the characteristic orthorhombic MFI structure of ZSM-5 (ICPDS, #PDF 44–0003)^5^ without framework collapse (Figure 1c). Moreover, no secondary phase such as AlF_*x*_ or Al_2_O_3_ appears in the PXRD patterns, confirming a well-prepared fluorinated ZSM-5 catalyst with structural and compositional integrity. At higher fluorination levels beyond the optimal range (1800 and 2400 mg g_zeolite_^–1^), decrease in diffraction intensity is observed, which, together with ICP results, indicates progressive framework deterioration associated with dealumination (X-Ray diffraction (XRD) results, Figure S2).

During catalytic reactions, the diffusion of molecules is strongly affected by the textural properties of catalysts, which can be readily modified by fluorination treatments.^25^ The textural properties of the prepared catalysts were characterized by N_2_ physisorption measurements, as shown in Figures 1d and S3. All F-ZSM-5 catalysts within the optimized NH_4_F range exhibited typical Type I isotherms, according to the IUPAC classification,^26^ featuring a steep N_2_ uptake at low relative pressure (p/p_0_ ≤ 0.04) corresponding to micropore filling (∼2 nm). With increasing NH_4_F content, the N_2_ adsorption amount slightly decreased, implying a minor reduction in micropore volume (Figure S4), which can be ascribed to partial dealumination or chemically bonded fluorine species (Al–F, etc.) by the fluorination, as supported by the ICP-OES and ^27^Al NMR results (*vide infra*). Meanwhile, this decrease in micropore volume is partially compensated by a subtle increase in mesopore volume at low fluorination levels (NH_4_F ≤ 600 mg g_zeolite_^–1^) (Table S1), indicative of mild framework etching rather than significant structural reconstruction.

In addition, all samples exhibited an H4-type hysteresis loop, which is attributed to the mesoporous features associated with interparticle voids of the catalysts.^5^ These results confirm that mild fluorination applied in this work caused only subtle textural changes while maintaining the intrinsic microporous framework of ZSM-5, as further supported by the scanning electron microscopy measurements (Figure S5) and high-resolution transmission electron microscopy (HRTEM) images (Figures 1e, andS6), with no visible structural deterioration and mesopore formation, in line with the PXRD and N_2_ physisorption results.

In contrast, previous studies have shown that harsh fluorination conditions can severely alter the zeolite texture to mesoporous properties.^27,28^ For example, Iadrat et. al.^27^ reported that harsh fluorination post-treatment could even induce microporosity in zeolites, as evidenced by the appearance of a typical H3-type hysteresis loop in the N_2_ adsorption–desorption isotherms. In our study, however, even at higher fluorination levels (F18 and F24-ZSM-5), no distinct hysteresis loop is observed (Figure S3), Indicating the absence of well-defined mesoporosity, as further supported by SEM images (Figure S5). Instead, a pronounced decrease in both microporosity and mesoporosity is evident (Table S1), suggesting that excessive fluorination (NH_4_ *F* > 600 mg g_zeolite_^–1^) leads to framework degradation and partial pore collapse rather than the formation of mesoporous networks. This behavior is consistent with previous observations by Qin et al., where such behavior was attributed to localized framework dissolution without the formation of continuous mesoporous networks.^29^

The role of fluorine in the acidic properties of ZSM-5 was characterized by NH_3_-temperature-programmed desorption (NH_3_-TPD) analysis, and the corresponding desorption profiles are shown in Figures 2a and S7. The pristine ZSM-5 and F-ZSM-5 catalysts exhibited two distinct NH_3_ desorption events: a low-temperature peak (*l-peak*) appearing at 150–300 °C and a high-temperature peak (*h*-peak) at 350–500 °C, which are commonly associated with weak and strong acid sites, respectively,^30,31^ with the former possibly involving non- or weakly acidic sites.^32^ The overall NH_3_ desorption amount increased under moderate fluorination and declined at higher NH_4_F loadings (>300 mg g_zeolite_^–1^). Quantitatively, the total acidity of pristine ZSM-5 (275 μmol g^–1^) increased to 350 μmol g^–1^ for F1-ZSM-5 and 360 μmol g^–1^ for F3-ZSM-5 (Figure 2b). However, a further increase in NH_4_F content reduced the acidity to 270 μmol g^–1^ for F6-ZSM-5, approaching that of pristine ZSM-5 catalyst, indicating partial loss of acid sites due to excessive fluorination.

**Figure 2:**
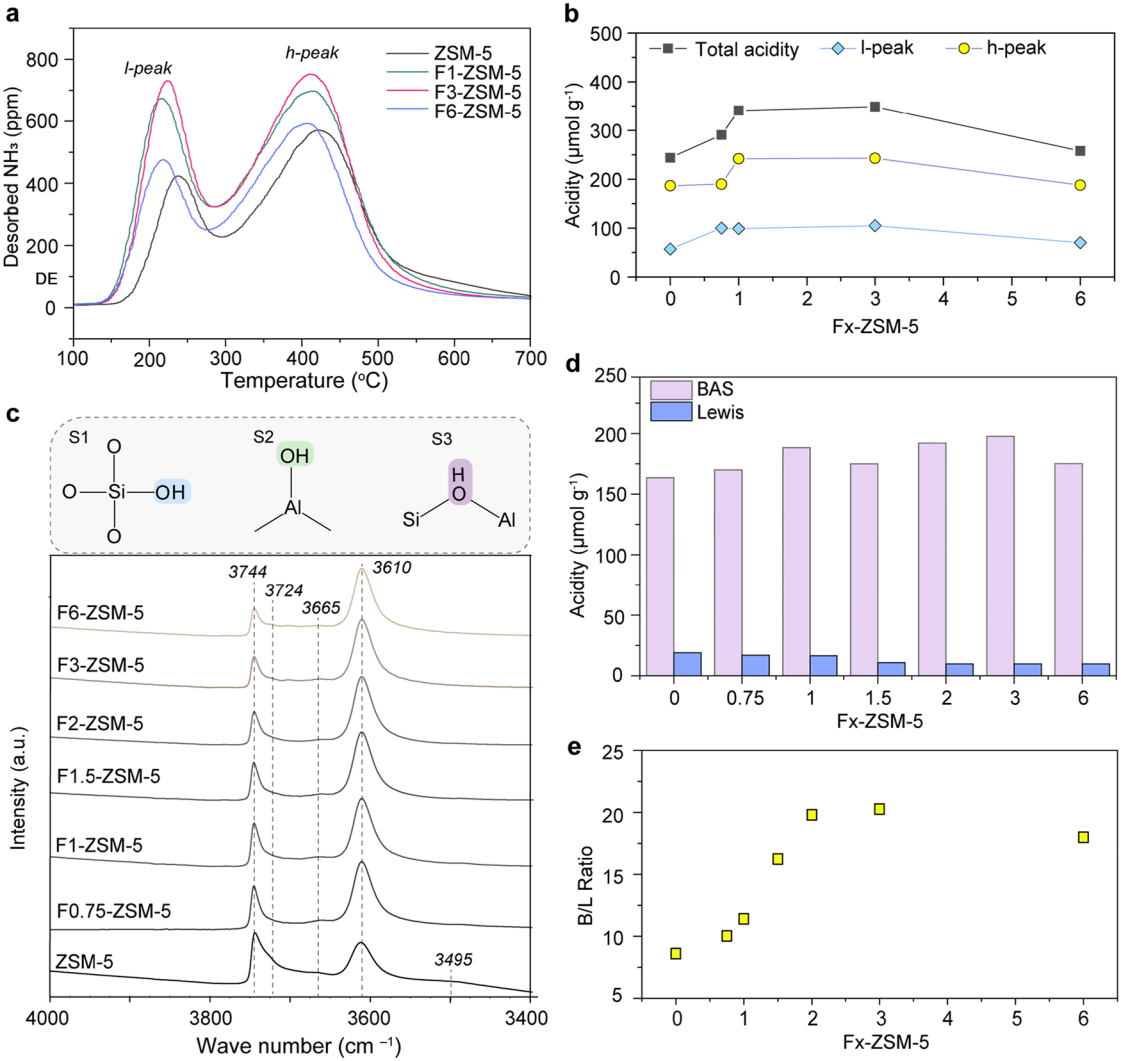
Acidity characterization of the prepared catalysts. (a) NH_3_-TPD profiles and (b) the calculated acidity of the parent ZSM-5 and F-ZSM-5 based on NH_3_-TPD results. (c) FTIR spectra of the framework of catalysts. (d) Pyridine-FTIR quantified BAS acidity and Lewis’s acidity. (e) Corresponding B/L ratios of catalysts of the catalysts as a function of fluorine content.

The attribution of the *l-peak* remains controversial, although it is generally assigned to weak acid sites. For example, Niwa et al.^32^ have suggested that this peak originates from the desorption of physically adsorbed NH_3_, whereas hydrogen-bonded NH_3_ on BASs within confined micropores may also contribute. Considering these uncertainties, the quantitative evolution of *h*-peak more reliably represents desorption from strong Brønsted acid sites (BASs).

The *h*-peak-associated acidity increased from 187 μmol g^–1^ for pristine ZSM-5 to 242 μmol g^–1^ for F1-ZSM-5 and 243 μmol g^–1^ for F3-ZSM-5, followed by a decline to 188 μmol g^–1^ for F6-ZSM-5. This behavior can be rationalized by two concurrent effects of fluorination: (i) generation of new acid sites, likely associated with F-polarized groups, and (ii) partial dealumination that reduces the number of intrinsic Si–OH–Al BASs in ZSM-5, as supported by the ICP results in Figure 1b and the NMR analysis discussed later (*vide infra*, Figure 3). Moreover, the *h*-peak position of pristine ZSM-5, centered at 427 °C, progressively shifted downward to 412 °C for F1-ZSM-5, 409 °C for F3-ZSM-5, and 403 °C for F6-ZSM-5, implying that the new acid sites generated upon fluorination possess lower acid strength than the intrinsic BASs in ZSM-5, as further corroborated by the NH_3_-TPD profile of the fluorinated silicalite-1 (Figure S8).

**Figure 3:**
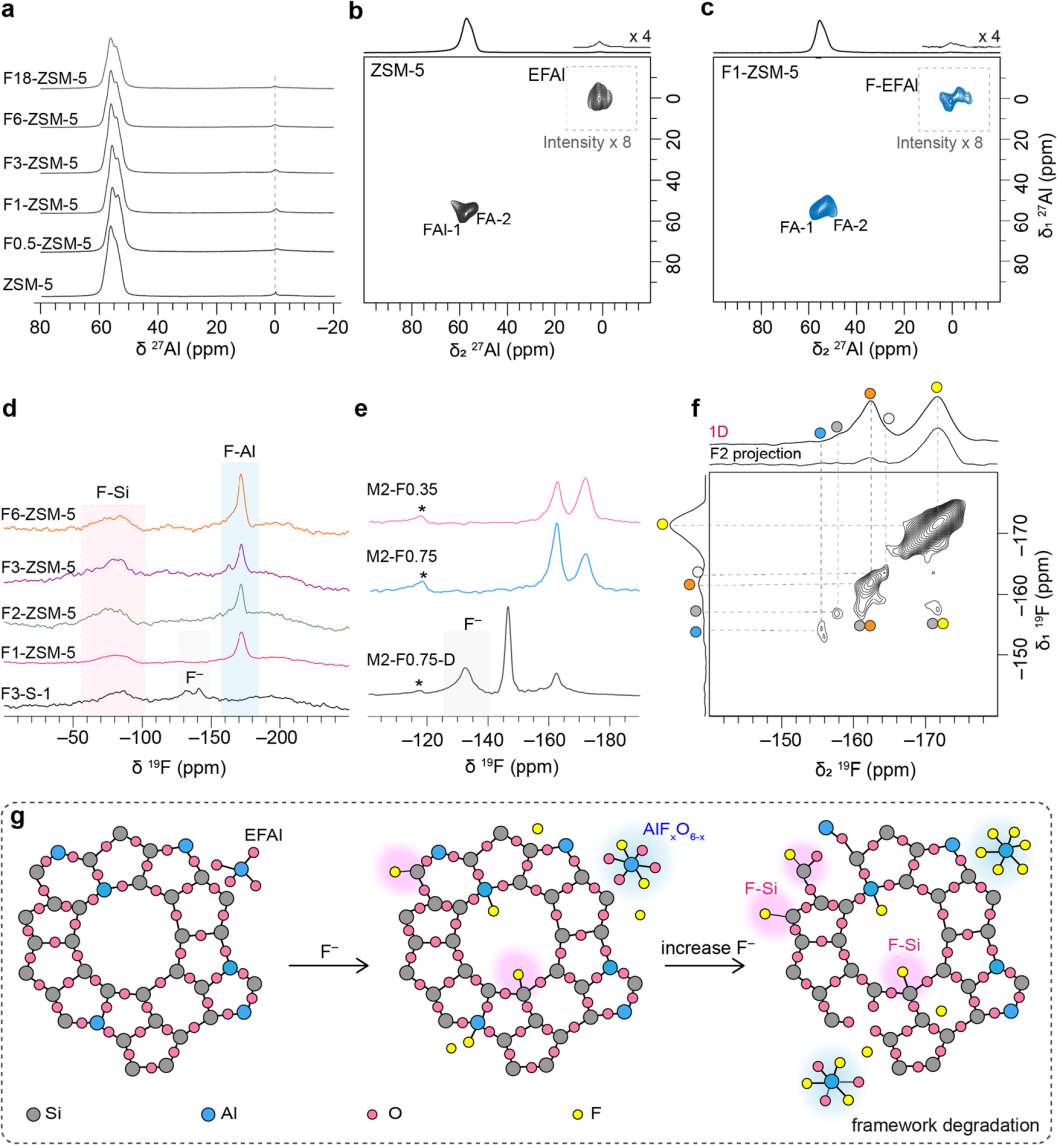
Solid-state NMR characterization of aluminum and fluorine environments in the catalysts. (a) 1D ^27^Al MAS NMR spectra of calcined parent ZSM-5 and F-ZSM-5 catalysts. ^27^Al 3QMAS spectra of (b) parent ZSM-5, and (c) F1-ZSM-5. ^19^F MAS NMR spectra of (d) F-ZSM-5 and F-silicalite-1, and (e) F0.35, and F0.75 -ZSM-5 prepared by methods 2 (M2). (f) 2D ^19^F–^19^F NOESY spectrum of F0.35-ZSM-5 (M2). (g) Schematic illustration of the proposed coordination environments of fluorine species in ZSM-5 under fluorination conditions.

To further elucidate the acidity evolution revealed by the NH_3_-TPD results, Fourier-transform infrared spectroscopy (FTIR) spectra of the parent and fluorinated ZSM-5 samples were recorded and normalized for direct comparison, as illustrated in Figure 2c. In zeolites, the bridging hydroxyl groups (Si–OH–Al) associated with BASs typically give rise to a characteristic O–H stretching vibration band near 3610 cm^–1^.^33,34^ In this study, this band is clearly observed for the parent ZSM-5, and its intensity increases after fluorination, suggesting an increased number of hydroxyl groups with comparable vibrational characteristics near the band at 3610 cm^–1^. However, ICP analysis revealed partial dealumination after fluorination, indicating that the increased intensity of the 3610 cm^–1^ band cannot be ascribed to the increase in framework Si–OH–Al groups. Instead, this enhancement most likely originates from the formation of fluorine-induced polarized hydroxyl species, as further supported by the changes observed for Si–OH groups (*vide infra*). These newly formed and polarized hydroxyls associated with Si–F interactions possess the BAS-like spectroscopic signature, with a stretching vibration red-shifted to 3610 cm^–1^, accounting for the enhanced acidity in the fluorinated ZSM-5 catalysts as observed in NH_3_-TPD results.

In addition to the 3610 cm^–1^ band, another distinct band at 3744 cm^–1^, corresponding to terminal Si–OH groups,^35,36^ is observed in all catalysts. In principle, the removal of framework Al is expected to increase the number of silanol groups.^37^ However, in contrast to this expectation, the intensity of this silanol band decreases progressively with increasing NH_4_F content, suggesting that fluorination consumes or transforms terminal silanol groups.

To verify the above hypothesis, pyridine adsorption FTIR spectroscopy was employed to identify the nature of acid sites. The adsorption bands at 1548 and 1454 cm^–1^ (Figure S9) correspond to pyridine adsorbed on BAS and LAS, respectively.^5,38^ The intensity of the BAS band increased when the fluoride content increased, while the LAS decreased. Quantitatively, the concentration of BAS in the parent ZSM-5 is 163.5 μmol g^–1^, which rises to 188.3 μmol g^–1^ in F1-ZSM-5, and 197.6 μmol g^–1^ in F3-ZSM-5, then declines to 175.2 μmol g^–1^ in F6-ZSM-5 (Figure 2d). It is worth noting that the NH_3_-TPD measurements may overestimate the acidity, as reported previously,^5^ yet both techniques reveal the same overall trend that F3-ZSM-5 exhibits the highest acidity, which decreases upon further NH_4_F increase.

In addition, the gradual decrease of LAS with increasing NH_4_F content at low loadings (≤200 mg g_zeolite_^–1^) can be attributed to the fluorinated extra-framework Al (EFAl) and removal by the subsequent DI water washing. With the further increase of NH_4_F, the LAS remained similar, which is ascribed to the formation of the EFAl during the calcination process. The B/L ratio, derived from the integrated intensities of the pyridine adsorption bands, provides a useful measure of the relative abundance of BAS and LAS (Figure 2e).^39,40^ Notably, the B/L ratio value increased from 7 in parent ZSM-5 to the highest B/L ratio of 21 in F3-ZSM-5, confirming the relative increase of BAS in fluorinated ZSM-5.

To gain structural insight into the origin of the acidity changes, ^27^Al solid-state nuclear magnetic resonance (NMR) spectroscopy was performed to examine the evolution of Al coordination upon fluorination (Figure 3a). It is well established that more than one type of framework Al (FAl) species exists in ZSM-5. Specifically, the resonances at 55.9 ppm (FAl-1) and 54.3 ppm (FAl-2) are assigned to crystallographically distinct tetrahedral FAl species: fully bonded FAl and partially bonded FAl, respectively.^5,41^ With increasing NH_4_F content, the overall intensity of the FAl signal gradually decreases, while the FAl-2 component becomes relatively more pronounced.

It is worth noting that Bruno et al. have shown that fluorine can direct the siting of Al atoms during hydrothermal synthesis when used as a mineralizing agent, leading to a preferential formation of specific framework Al environments (corresponding to the resonance near 55.9 ppm, assigned to FAl-1 in this work) and thereby influencing the zeolite acidity.^42^ Conversely, in the present study, the FAl-2 component is preferentially enhanced rather than FAl-1, suggesting that the acidity changes observed here are not governed by fluorine-induced Al siting effects. This difference is reasonable, considering the distinct postmodification fluorination route employed here, followed by the removal of fluorine-induced mobile Al species (e.g., AlFx groups, Figure 3g) through washing with DI water. Accordingly, FAl-2 is more plausibly attributed to partially bonded FAl species, in line with the interpretation proposed by Jeffery and co-workers.^43^ This evolution indicates a progressive partial transition from fully bonded to partially bonded FAl sites (e.g., F–FAl, as corroborated by ^19^F NMR results, *vide infra*), eventually leading to partial zeolite dealumination, as supported by the ICP and XRD results (Figure 1b,c). Consequently, fluorination reduces the number of intrinsic Si–OH–Al BASs in ZSM-5, whereas the overall enhancement of acidity after moderate fluorination can be ascribed to the fluorine-induced polarized hydroxyls.

In addition, the resonance corresponding to extra-framework Al (EFAl) near 0 ppm does not show visible growth.^41,44^ Instead, its intensity slightly decreases at higher fluorination levels (NH_4_F ≥ 300 mg g_zeolite_^–1^, *x* ≥ 3), which can be attributed to the formation of soluble AlFx species and their subsequent removal during DI water washing. Moreover, a slight broadening of the EFAl resonance near 0 ppm is observed after fluorination, indicating an increase in the heterogeneity of EFAl environments.^5^ This broadening likely arises from the coexistence of multiple octahedral Al species with varying degrees of F/OH coordination, leading to enhanced quadrupolar interactions and resulting in a broader distribution of electric field gradients.

To clearly differentiate the various Al species by eliminating the second-order quadrupolar broadening associated with half-integer nuclei, ^27^Al triple-quantum magic angle spinning (3QMAS) experiments were conducted on the parent ZSM-5 and F1-ZSM-5 samples (Figure 3b,c), and multiple Al species with different chemical speciation can be found. The signals at 57.0 and 55.7 ppm correspond to the FAl-1 and FAl-2 sites observed in the one-dimensional (1D) ^27^Al NMR, respectively. With the fluorination, the symmetry of the surroundings of Al was changed, and the ^27^Al resonance of FAl-2 shifted from diagonal to quadrupolar-induced shifts. This indicates that the electronic environment of FAl sites is highly distorted,^5,45^ due to its interaction with fluorine, as supported by ^19^F NMR results (*vide infra*). In addition to the intrinsic EFAl resonance at 2.0 ppm in the parent ZSM-5, new resonances at 0 ppm and −0.5 ppm emerges after fluorination. These are assigned to F-coordinated EFAl species (AlO_X_F_Y_ coordination variants), as further evidenced by the characteristic of ^19^F NMR signatures (*vide infra*).

Guided by the acidity profiling and ^27^Al NMR evidence that fluorination induces dealumination and perturbs Al coordination, we next employed ^19^F MAS NMR to probe the coordination states and local environments of fluorine in the fluorinated samples (Figure 3d). A sharp resonance region (blue) contains two distinguishable peaks at −163 and −172 ppm, which are assigned to F–EFAl^46,47^ and F–FAl^46,48^ species, respectively. Notably, these two contributions become more evident in samples treated with NH_4_F amounts higher than 100 mg g_zeolite_^–1^ (*x* > 1), while their resolution is attributed to be further modulated by the coexistence of multiple AlO_X_F_Y_ coordination variants, as supported by the ^27^Al MAS NMR results. To further strengthen this assignment, two selectively fluorinated samples (defined as M2-F0.35 and M2-F0.75) with a significantly higher fluorine content but not subjected to DI-water washing were measured and are plotted in Figure 3e. Notably, two distinguishable resonances are consistently observed across the fluorinated-ZSM-5 catalysts (Figure 3d). In particular, the M2-F0.75 sample with a higher fluorination level exhibits a markedly enhanced resonance at −163 ppm (F–EFAl species), relative to that at −172 ppm (F-FAl). This suggests that the higher fluorine loading promotes the formation of F-EFAl coordination states, thereby corroborating the above ^27^Al and ^19^F spectral assignments.

Accurate quantification of fluorine in zeolitic materials remains highly challenging because fluorine is a light, nonmetallic element and the zeolite matrix imposes additional analytical constraints.^49^ Reliable bulk analysis typically requires HF-based digestion of the siliceous framework, which inherently contains fluorine and complicates the quantification. In our case, attempts using nondestructive X-ray-based techniques such as X-ray Photoelectron and Energy Dispersive X-ray Spectroscopy (XPS) did not provide reliable quantitative results due to low sensitivity and pronounced matrix effects.^50^ Nevertheless, the ^19^F NMR spectra clearly confirm fluorine incorporation, showing a systematic increase in resonance intensity with increasing NH_4_F dosage, as shown in Figures 3d andS10.

Given that multiple fluorine species exist in F-ZSM-5, to assess spatial relationships among fluorine species and to obtain more detailed insights into their local chemical environment, 2D ^19^F–^19^F nuclear Overhauser effect spectroscopy (NOESY) MAS NMR analysis was employed. The efficiency of NOESY relies on dipolar-driven cross-relaxation, which generally requires finite mixing times. When the spin density is low or transverse relaxation is relatively fast, cross peaks are intrinsically weak.^51,52^ To overcome this, we performed the 2D ^19^F–^19^F NOESY MAS NMR on selectively prepared samples F0.35-ZSM-5 (M2) containing higher fluorine content while preserving the same fluorine species distribution based on the 1D ^19^F NMR resonance spectra (Figure 3f), thereby increasing the number of magnetization-transfer pathways and the observable cross-peak intensities.

The diagonal of Figure 3f displays two principal high-field resonances at −163 and −172 ppm, matching the 1D assignments of F–EFAl and F–FAl, respectively.^46^ Notably, weak correlations involving a shoulder near −156 and −158 ppm, together with their high-field position and NOESY cross-peaks to the −163 and −172 ppm signals, suggest secondary Al-bound fluorine sites, with chemically inequivalent Al-bound fluorine (AlO_X_F_Y_ coordination variants).^53^ This interpretation is consistent with our ^27^Al NMR observations above, which showed that fluorine perturbs both EFAl species (e.g., giving rise to multiple resonances at 0 ppm and – 0.5 ppm) and FAl sites in ZSM-5.

Beyond the F–Al coordination states, a broad ^19^F resonance between −60 and −110 ppm is observed (pink region in Figure 3d), commonly assigned to fluorine bound to framework Si (F–Si) in MFI silicalite.^54^ This attribution is corroborated by the fluorinated silicalite-1 reference (F3–S-1, Figure 3d), which exhibits a resonance only in this F–Si region (pink) and lacks the high-field F–Al signal (blue), thereby confirming our above F–Si and F–Al assignments. This assignment is further supported by XPS analysis, which shows shifts of the Al 2p and Si 2p peaks toward higher binding energies upon fluorination, consistent with the electronic perturbation of the zeolite framework by fluorine species (Figures S11, S12, and S13), suggesting that the enhanced acidity originates from fluorine-induced interactions with both Al and Si environments.

Additionally, an intermediate feature between −120 to −150 ppm is observed, which is pronounced in the dried fluorinated sample before calcination (M2-F0.75-D) (Figure 3e) and is attributed to fluoride species trapped within the pores.^55^ In the absence of Al, the fluoride is insufficiently consumed and therefore remains partially trapped. Although such trapped fluoride species are not directly involved in our Al-containing ZSM-5 system, this observation nonetheless offers complementary insight, further enriching our understanding of how fluorine evolves and manifests in different chemical environments within zeolites.

Previous ^19^F and ^27^Al MAS NMR analyses have demonstrated that fluorine interacts with the zeolite framework, altering both aluminum and silicon environments. Therefore, to further investigate the local silicon coordination environments and their response to fluorination, ^29^Si MAS NMR spectroscopy was employed (Figure 4). In the single-pulse ^29^Si MAS spectra, both the parent ZSM-5 and fluorinated ZSM-5 (F3-ZSM-5) exhibit a dominant resonance at −114 ppm, characteristic of Q^4^ (Si­(OSi)_4_) species (Q^n^ denotes a Si atom bonded via n Si–O–Si bridges) in the MFI framework.^56,57^ A minor shoulder at – 107 ppm is discernible in both samples and is attributed to Q^3^ (Si­(OSi)_3_X, where X represents a terminal group) sites.^58,59^ Importantly, no appreciable increase in Q^2^ (Si­(OSi)_2_X_2_) signals is detected in either sample, indicating that the zeolite framework remains well-preserved upon fluorination. This observation is consistent with XRD and N_2_ physisorption analyses, which confirm that the crystallinity and microporosity of zeolites are well-retained.

**Figure 4:**
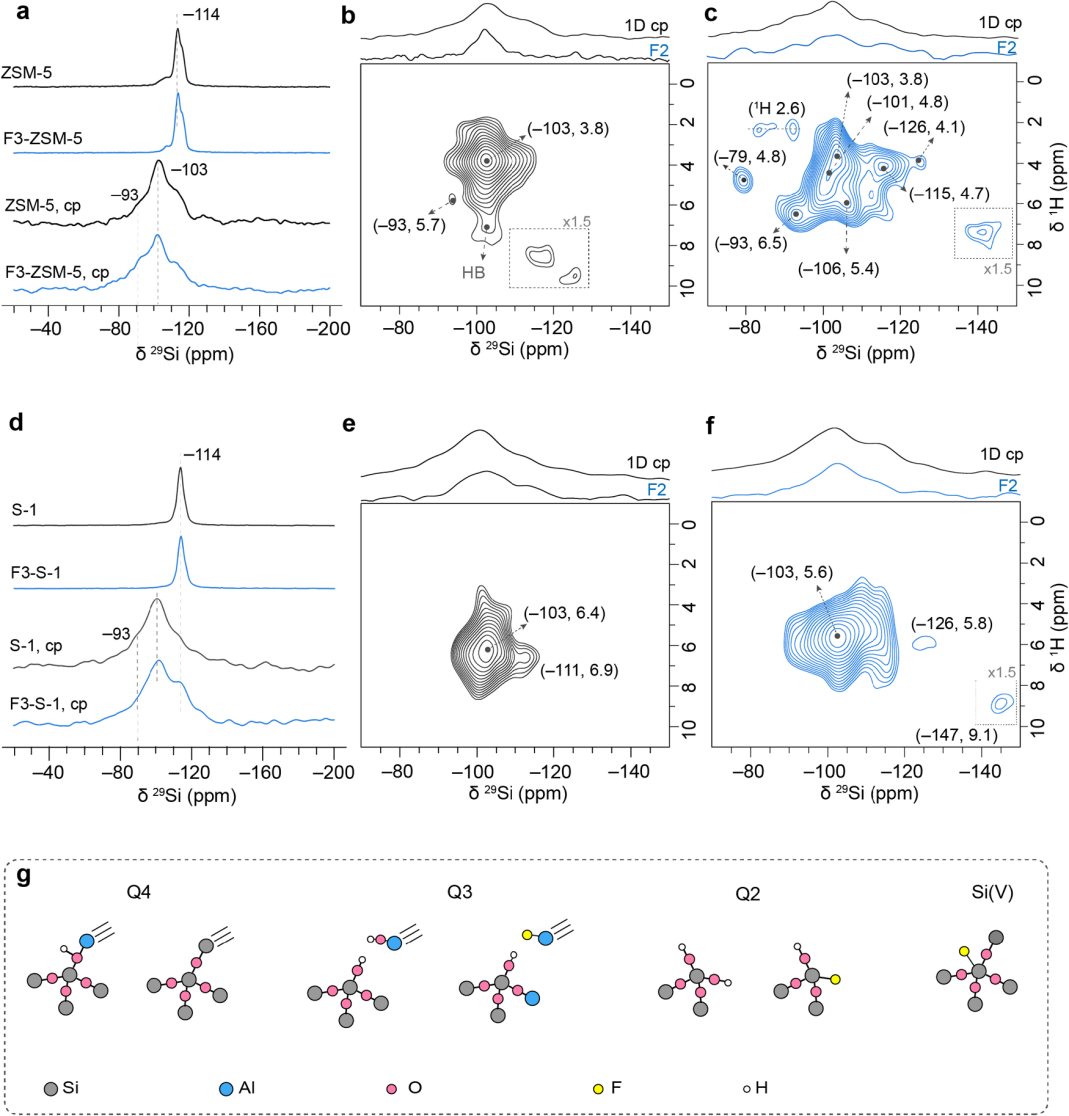
Solid-state NMR characterization of silicon environments in catalysts. Direct ^29^Si and ^1^H–^29^Si CP MAS spectra of calcined (a) parent ZSM-5 and F3-ZSM-5, and calcined (d) pristine silicalite-1 and F3-silicalite-1. 2D ^1^H–^29^Si HETCOR a spectra of (b) parent ZSM-5, (c) F3-ZSM-5, (e) silicalite-1 and (f) F3-silicalite-1. (g) Schematic illustration of the proposed silicon environments in fluorinated ZSM-5.

Due to the coexistence of multiple silicon environments and the dominance of Q^4^ species in the ZSM-5, only limited structural information can be obtained from direct-excitation single-pulse ^29^Si MAS experiments (Figure 4a). Subtle variations associated with silanol nests or proton-associated defective species are difficult to resolve under these conditions. To obtain deeper insights into the silicon chemistry, particularly the evolution of Q^3^ and Q^2^ species, short-contact ^1^H–^29^Si cross-polarization (CP) measurements (Figure 4a) were therefore carried out. In contrast to the parent ZSM-5, the F3-ZSM-5 sample exhibits enhanced CP intensity in the Q^3^ (−103 ppm)^60^ and Q^2^ (−93 ppm)^61^ resonance, while the CP intensity of the Q^4^ resonance (−114 ppm)^57,61^ shows a modest decrease. This redistribution of CP intensity from Q^4^ to Q^3^ and Q^2^ species is attributed to the fluorination-induced dealumination of the zeolite framework, in agreement with the ^27^Al MAS NMR results and ICP results.

To overcome the limitations of 1D CP spectra and directly correlate protons with specific silicon sites, 2D heteronuclear ^1^H–^29^Si correlation (HETCOR) measurements were carried out (Figure 4b,c). In the parent ZSM-5 (Figure 4b), a dominant correlation (^1^H, ^29^Si) at (−103, 3.8) is observed, corresponding to Q^3^ silicon linked with BASs.^62^ This correlation may also include minor contributions from hydrogen-bonded silanol species,^59^ such contributions are expected to be limited as further supported by the 2D ^1^H–^29^Si HECTOR results (Figure 4e, *vide infra*). Although literature reported that BAS can shift downfield toward near 6 ppm when involved in hydrogen bonding, forming so-called polarized BASs.^57,63^ Consistent with this, a weak correlation at (−93, 5.7) is observed and is tentatively assigned to hydrogen-bonded BASs.^63^ Furthermore, several weak cross peaks emerge in the highly downfield proton region (7–10 ppm), correlated with ^29^Si chemical shifts ranging from −90 to −130 ppm. These features are attributed to strongly hydrogen-bonded silanol groups and silanol nests located at framework defects or pore intersections.^58^

Upon fluorination, significant changes are observed in the 2D ^1^H–^29^Si HETCOR spectra of F3-ZSM-5 (Figure 4c). The original cross peak (^29^Si, ^1^H) at (−103, 3.8) becomes markedly weaker, accompanied by the emergence of multiple new silicon environments. Notably, more abundant Q^3^ species appear at −101, and −106 ppm, correlating with ^1^H resonances at 4.8 and 5.4 ppm, respectively. The correlation at (−106, 5.4) is assumed to be the polarized BAS with the Si­(OSi)_3_(OAl). The correlation peaks at (−93, 6.5), which is assumed to be originally polarized cross peak at (−93, 5.7) because of the presence of fluorine. These correlations suggest the presence of polarized BASs or hydrogen-bonded BAS situated on Q^3^ silicon, in agreement with the ^1^H MAS NMR data discussed later (*vide infra*).

In addition to the evolution of Q^3^-BAS-realated correlations, fluorination also gives rise to new ^1^H–^29^Si cross peaks in the Q^2^ region at (−84, 2.6) and (−79, 4.8) (Figure 4c), originating from fluorination-induced dealumination, and consequent formation of silanol nests grouped with polarized silanol groups. Although proton resonance near 2–3 ppm in ZSM-5 is often assigned to hydroxyls associated with EFAl (EFAl–OH),^64,65^ this assignment is unlikely in the present system. First, ^27^Al MAS NMR does not show any appreciable increase in EFAl species (Figure 3c). Besides, the well-resolved correlation at (−84, 2.6) suggests the spatial proximity between the ^1^H and ^29^Si rather than to EFAl–OH species. This resonance is therefore assigned to polarized Si–OH groups, as corroborated by the ^1^H NMR results discussed later (*vide infra*). In addition, the cross peak at (−79, 4.8) reflects Q^2^ silicon in a more deshielded ^29^Si resonance than the – 84 Q^2^ site, suggesting that fluoride is likely interacting with or coordinated to these Q^2^ silicon sites, inducing more polarized protons with a resonance at 4.8 ppm.

Unlike the parent ZSM-5, the F-modified sample displays distinct Q^4^ correlations with ^1^H in the 2D ^1^H–^29^Si HETCOR spectra as well as signals from more upfield silicon environments. For instance, the cross peak at (−115, 4.7) corresponds to Q^4^-type silicon sites in close spatial proximity to polarized hydroxyl protons (^1^H at 4.7 ppm), suggesting that fluorination generates new proton-containing species located near Q^4^ silicon sites. These protons are assumed to originate from silanol groups^65^ formed upon dealumination, which produce adjacent silanol nests or defect-associated Si–OH groups that become further polarized by fluorine, their proximity to intact Q^4^ silicon sites enables the emergence of dipolar-mediated ^1^H–^29^Si correlations at (−115, 4.7).

Beyond the Q^4^ region, additional correlations between −126 and −145 ppm serve as fingerprints of five-coordinated silicon bonded with fluoride (Si­(O)_4_F^–^).^56^ As reported by Koller et.al., the resonance near – 125 ppm arises from the dynamic exchange between four- and five-coordinate silicon centers, whereas the signal near −145 ppm is characteristic of static five-coordinate Si­(O)_4_F^–^ units (as illustrated in Figure 4g).^56^ Strikingly, analogous features at −126 and the signal assigned to five-coordinate Si­(O)_4_F^–^ (−147 ppm) are detected in the fluorinated silicalite-1 (Figure 4f, *vide infra*), consistent with the ^19^F MAS NMR results. Taken together, these results establish that fluorination universally drives the formation of covalent Si–F linkages and five-coordinate Si­(O)_4_F^–^ species, representing a fundamental restructuring pathway of siliceous frameworks under fluorination treatment.^56^

Building on this evidence, the fluorinated silicalite-1 further illustrates the structural response of a purely siliceous framework to fluorination (Figure 4d). In 2D ^1^H–^29^Si HETCOR spectra, the formation of a resonance peak at (−126, 5.8) and (−147, 9.1) suggested the polarized hydroxyl protons in close spatial proximity to the dynamically exchanging four-/five-coordinate silicon and the static five-coordinate (Si­(O)_4_F^–^) centers, in agreement with our above assignments. Beyond establishing these F-induced Si coordination states, the silicalite-1 system also offers a complementary perspective accompanying framework-level response. The ^29^Si MAS NMR spectra show no evidence of Q^2^-type defect formation; instead, the Q^4^ resonance intensity increases, suggesting that NH_4_F treatment partially heals the siliceous silanol nest defects.^66^ This effect is not pronounced in the ZSM-5 system due to the competing dealumination pathway, yet it provides valuable insight into the multifaceted role of fluorine in modulating zeolitic frameworks. Consistent with this interpretation, the major ^1^H–^29^Si correlations in pristine silicalite-1 appear ∼ 6.4 ppm (Figure 4e), whereas those in F3–S-1 (Figure 4f) resonate ∼5.8 ppm, attributing to the reduced population of strongly hydrogen-bonded silanol nests following defect healing, in agreement with the ^1^H MAS NMR results (Figure S14, *vide infra)* and XRD results (Figure S15).

As revealed by the ^1^H–^29^Si HETCOR spectra, fluorination leads to a more diversified proton environment surrounding silicon atoms. To further elucidate the resulting acidity evolution in terms of changes in protonic species and framework chemistry, solid-state ^1^H MAS NMR spectroscopy was employed. The 1D ^1^H MAS spectra provide direct information on the types and strengths of hydroxyl groups in the parent ZSM-5 and F-ZSM-5 samples (Figure 5). In Figure 5a, the parent ZSM-5 displays two distinct resonance signals at 1.6 and 3.9 ppm, corresponding to terminal silanol groups (Si–OH) and BASs (Si–OH–Al), respectively.^65,67^ It is worth noting that silanol-related signals (∼1.6 ppm) are absent in the 2D ^1^H–^29^Si HETCOR spectra across all samples, likely due to the weak dipolar coupling and dynamic nature of isolated silanol groups, which can lead to partial averaging of dipolar interactions and reduced cross-polarization efficiency.^68–70^

**Figure 5:**
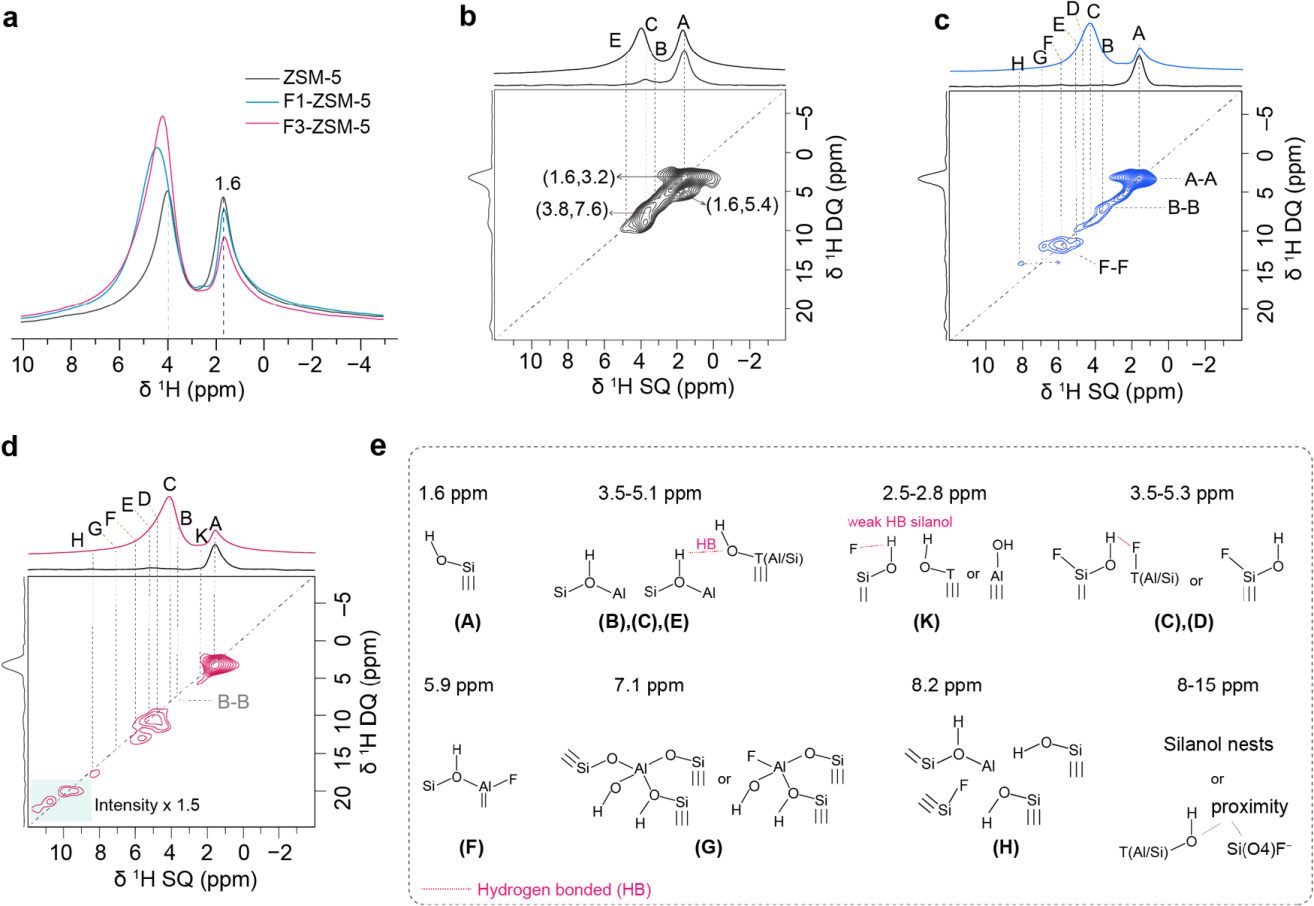
Solid-state NMR analysis of proton environments in ZSM-5 and F-ZSM-5 catalysts. (a) Direct ^1^H MAS NMR spectra of parent ZSM-5, F1-ZSM-5, and F3-ZSM-5. 2D ^1^H DQ–SQ MAS NMR spectra of (b) parent ZSM-5, (c) F1-ZSM-5, and (d) F3-ZSM-5. (e) Schematic illustration of the proposed proton environments in fluorinated ZSM-5.

Upon fluorination, the F1-ZSM-5 sample exhibits a decrease in the intensity of the 1.6 ppm signal, which further diminishes with increasing NH_4_F content up to 300 mg g_zeolite_^–1^ (F3-ZSM-5), indicating a progressive reduction of intrinsic terminal silanol groups in ZSM-5. Meanwhile, the resonance associated with BASs at ∼ 3.9 ppm shows a significant enhancement and broadening toward the lower field region (Figure 5a). This broadening feature, extending up to ∼10 ppm, suggests the emergence of additional proton species exhibiting BAS-like characteristics^71^ or located in a more deshielded environment.^59^ Such downfield-shifted resonances are indicative of stronger hydrogen bonding or increased proton acidity,^71^ likely arising from fluorine-induced polarization of framework hydroxyls (BASs, and Si–OH). The evolution of these proton species aligns with the FTIR observations (Figure 2c). The attenuation of the 3745 cm^–1^ band agrees with the loss of terminal silanol, while the intensified and red-shifted 3610 cm^–1^ band corresponds to the strengthened BAS-like OH groups. Together, the enhanced acidity of ZSM-5 due to the fluorination can be further attributed to these protons, as observed in both NH_3_-TPD and pyridine FTIR results.

The broadening and enhanced intensities of the BAS-like proton signal (left peak at ∼3.9 ppm) in ZSM-5 (Figure 5a) upon the fluorination suggests the coexistence of multiple protonic environments. To further probe the spatial proximity and interaction among different hydroxyl species and separate overlapping hydroxyl resonances unresolved in the 1D spectrum, 2D ^1^H double quantum–single quantum (DQ–SQ) MAS NMR experiments were performed (Figure 5b**–**d). In parent ZSM-5, consistent with the 1D result, a strong diagonal correlation signal at (1.6, 3.2) is assigned to the autocorrelations of terminal silanols (A). A cross-correlation at (1.6, 5.4) is attributed to the spatial proximity between terminal silanol and BAS protons (∼3.8 ppm, signal C), forming a weak hydrogen bond.

Within the BAS region, a prominent diagonal correlation at (3.8, 7.6) corresponds to framework BASs protons at 3.8 ppm (C), with a weak hydrogen-bonded interaction with surrounding protons as mentioned above, which is dominant among BASs protons, as supported by the ^1^H–^29^Si HETCOR results (Figure 4b). Besides, a weaker resonance at 3.5 ppm (B) is observed, which can be assigned to BASs located in a less or non-hydrogen-bonded environment. In addition, a downfield correlation at (3.8, 9.2) originates from dipolar coupling between BAS at ∼3.8 ppm and a more deshielded, hydrogen-bonded BAS at ∼5.4 ppm (E).^72^ Indicating multiple BAS protons in parent ZSM-5, ranging from non-hydrogen-bonded to strongly hydrogen-bonded states.^73^

Upon fluorination, notable changes in the 2D ^1^H DQ–SQ MAS NMR spectrum were observed, as shown in Figure 5b,c. Compared with the pristine ZSM-5 (Figure 5b), the autocorrelation associated with terminal silanol (A) at (1.6, 3.2) progressively decreased with increasing NH_4_F content from 100 mg g_zeolite_^–1^ (F1-ZSM-5, Figure 5c) to 300 mg g_zeolite_^–1^ (F3-ZSM-5, Figure 5d), in good agreement with the observations in FTIR and 1D ^1^H MAS NMR results.

More pronounced changes occurred in the correlations corresponding to BAS’s protons. The diagonal autocorrelations of BASs at (3.5, 7.0) and (3.8, 7.6) were both reduced in F1-ZSM-5. With further increase of NH_4_F content to 300 mg g_zeolite_^–1^ (F3-ZSM-5, Figure 5d), the autocorrelation of signal B (∼3.5 ppm) disappeared completely, and was followed by the emergence of new downfield autocorrelation at (5.4, 10.8) (E), indicative of highly polarized or strongly hydrogen-bonded BASs because of the introduction of fluorine. Taken together, the evolution of BASs can be attributed to the fluorine-induced polarization of BASs and partial removal of framework Al, as discussed in the ^27^Al NMR results, suggesting that fluorine preferentially affects framework regions associated with FAl in the ZSM-5 system, where the Al–O–Si bonds are relatively more labile compared to Si–O–Si linkages.^74^

In addition to the intrinsic correlations observed in the parent ZSM-5, several new resonances emerged in the fluorinated samples. The signals at 5.9 ppm (F) and 7.1 ppm (G) are attributed to the BASs located in proximity to fluorine species (Figure 5e), likely representing transient intermediates formed during the fluorination-induced dealumination process. As the fluorination severity increased, these resonances gradually disappeared. Meanwhile, several isolated resonance islands appear in the 8–10 ppm region (Figure 5d) upon the severe fluorination corresponding to highly deshielded protons, which are assigned to strongly hydrogen-bonded hydroxyls with fluorine and silanol nests generated by the removal of framework Al, consistent with 1D ^1^H MAS NMR results.

Interestingly, a distinct diagonal autocorrelation signal at ∼2.5 ppm (K) was observed in the 2D ^1^H DQ–SQ MAS spectrum of F3-ZSM-5 (Figure 5d). Although such a resonance is often attributed to hydroxyl groups associated with EFAl (EFAl–OH),^65^ such an assignment does not align with our observations. The ^27^Al MAS NMR spectra of fluorinated ZSM-5 showed no indication of increased EFAl formation, suggesting that the ∼ 2.5 signal in F3-ZSM-5 arises from polarized hydroxyl groups owing to fluorine. This interpretation is further supported by the ^1^H MAS NMR spectrum of fluorinated silicalite-1 (Figure S14), where an identical feature appears in the absence of aluminum, confirming that this feature originates from fluorine-polarized silanol groups rather than EFAl–OH species.

Moreover, the fluorination process creates multiple polarized silanol groups (∼2.5 to ∼6 ppm, Figure S14). These polarized silanol species collectively give rise to the measurable acidity of fluorinated silicalite-1, as reflected by the NH_3_-TPD desorption features (Figure S8, broadened desorption peak). Taken together, the increased intensity of the BAS-like resonance (∼3.9 ppm peak, with broadening toward higher chemical shift) in the 1D ^1^H MAS NMR spectrum and the O–H stretching vibration band near 3610 cm^–1^ are attributed to overlapping contributions from polarized silanol groups (Figure S14b), while the downfield broadening is attributed to the formation of stronger hydrogen-bonded BASs induced by fluorination. Last, as a side remark, the autocorrelation peaks of silanol-nest signals (Figure S14b) observed in pristine silicalite-1 disappear upon fluorination in the ^1^H DQ–SQ spectrum (Figure S14c,d), indicative of partial defect healing in silicalite-1, in agreement with the ^29^Si NMR results (Figure 4d–f).

Fluorination on ZSM-5 reduces the number of conventional BAS while simultaneously generating BAS-like sites through polarizing silanol groups. To assess how these acidity modifications impact catalytic performance, the methanol-to-hydrocarbon (MTH) reaction was employed as a probe and carried out at 400 °C with a WHSV of 16 h^–1^ (Figures 6, and S16). As shown in Figure 6a, the catalyst lifetime, defined as the onset of catalyst deactivation, increased markedly upon fluorination. F0.5-ZSM-5 exhibited an extended lifetime of 32.5 h compared with the parent ZSM-5 (22 h), and the maximum lifetime of 39.0 h was achieved for F1-ZSM-5, representing a 77% improvement over parent ZSM-5. The results suggest that catalyst lifetime can be enhanced by modifying the nature of the BAS, regardless of the total number of BAS. Although dealumination is generally known to prolong catalyst lifetime by reducing the BAS acidity, fluorination in the present system simultaneously introduces new BAS-like sites. The lifetime enhancement observed here cannot be explained solely by the loss of Al, as will be further corroborated by the product distribution and spent-catalyst analysis (Figure 6b–e, *vide infra*). Catalytic performance tests of F-silicalite-1 in different bed configurations demonstrate that polarized hydroxyl groups alone are insufficient to initiate and sustain the catalytic reaction (Figures S16 and S17). Therefore, we postulate that the observed changes in catalytic performance arise from a synergistic effect between intrinsic BASs and polarized hydroxyl groups.

**Figure 6:**
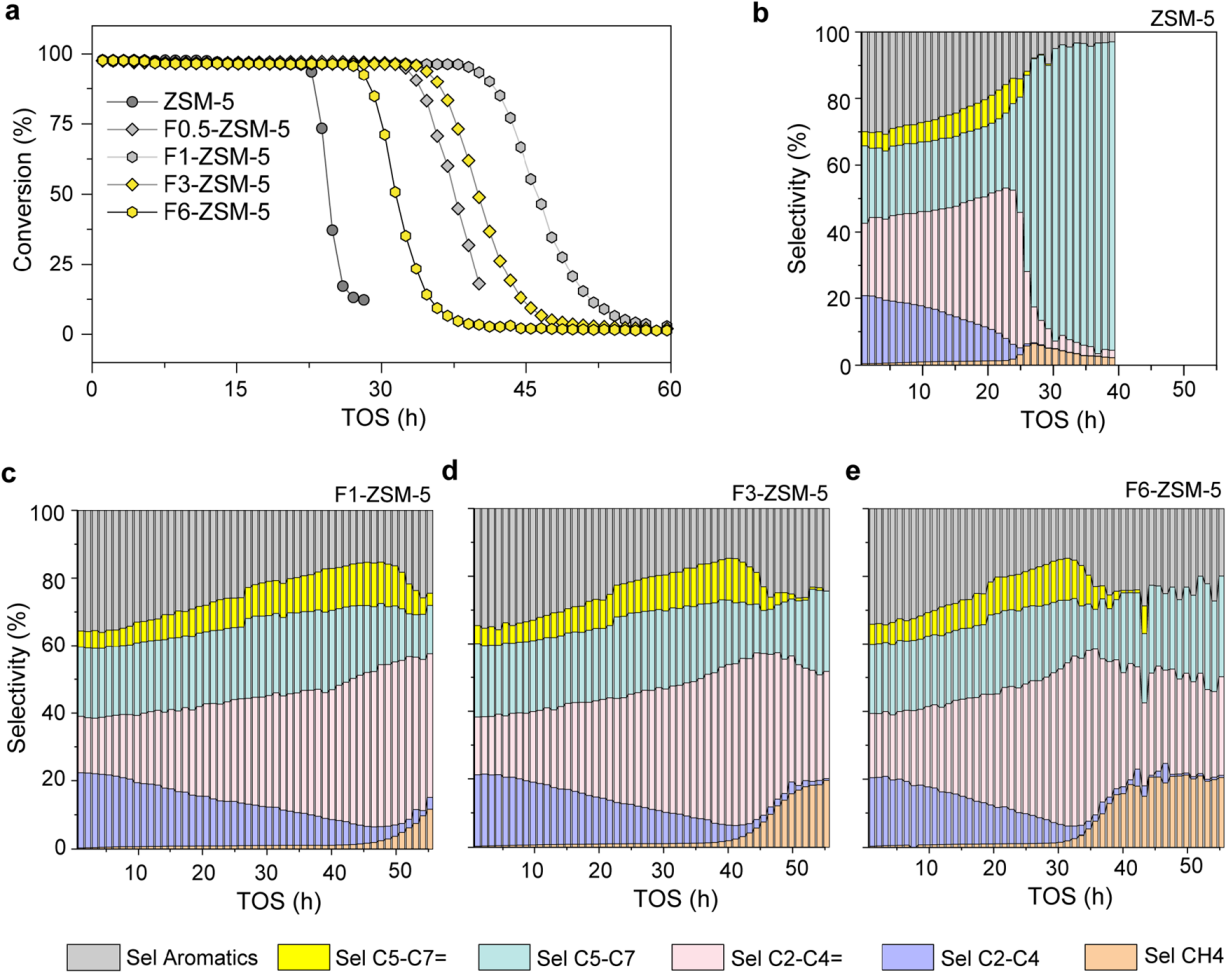
Catalytic activity of prepared catalysts. (a) Catalytic performance of ZSM-5 and F-ZSM-5 catalysts at 400 °C with a WHSV of 16 h^–1^. Product distributions of (b) parent ZSM-5, (c) F1-ZSM-5, (d) F3-ZSM-5, and (e) F6-ZSM-5.

However, further increasing the NH_4_F amount to 300 mg g_zeolite_^–1^ (F3-ZSM-5) reduced the lifetime to 34.6 h, and an even shorter lifetimes observed for F6-ZSM-5 (29 h), and F18-ZSM-5 (9 h, Figure S18). This decline at higher fluorination levels is attributed to fluorination beyond the optimal range, which induces increased dealumination and results in weakening of the zeolite framework. This is further supported by the ^27^Al 3QMAS NMR spectra (Figure S19) of the spent catalysts, where a significantly altered coordination environment of framework Al is observed in F18-ZSM-5, in contrast to the well-preserved framework Al signals in ZSM-5 and F1-ZSM-5.

To gain deeper insight into how fluorination influences catalytic performance, the evolution of product distributions over different catalysts was compared (Figure 6b–e). An enhanced selectivity toward aromatics was observed upon fluorination. For instance, F1-ZSM-5 exhibited a slightly higher aromatic selectivity (31.5%) than the parent ZSM-5 (30.4%) at 2 h time-on-stream (TOS). When the NH_4_F content increased to 300 mg g_zeolite_^–1^ (F3-ZSM-5, Figure 6d), the aromatic selectivity reached a maximum of 35.7% and it decreased to 34.2% with the fluorine further increased to 600 mg g_zeolite_^–1^ (F6-ZSM-5, Figure 6e). This trend mirrors the evolution of total acidity density observed in the NH_3_-TPD results (Figure 2a), implying that the newly formed polarized BAS-like hydroxyls are involved in MTH pathways, contributing to aromatic production. Given the close relationship between aromatic formation and hydrogen transfer behavior in MTH,^75,76^ the hydrogen transfer index (HTI, butanes/(butanes + butenes), Table S2) was evaluated.^77^ The parent ZSM-5 exhibits an HTI value of 0.595, which increases to 0.611 for F1-ZSM-5 and 0.630 for F3-ZSM-5. Upon increasing the NH_4_F content to 600 mg g_zeolite_^–1^ (F6-ZSM-5), the HTI decreases to 0.600. Consistently, a similar trend is observed for the paraffin-to-olefin ratios (PTO, C_2_–C_7_, Table S2), which serve as an additional indicator of overall hydrogen transfer activity.^78–80^ The PTO values of fluorinated ZSM-5 samples are generally higher than that of the parent ZSM-5 (1.46), reaching 1.92 for F3-ZSM-5 and remaining at 1.64 for F6-ZSM-5. These consistent trends suggests that moderate fluorination (≤600 mg g_zeolite_^–1^) enhances the hydrogen transfer activity, attributing the enhanced acidity density, whereas excessive fluorination (>600 mg g_zeolite_^–1^) leads to framework degradation and loss of functionality (Figure S19, Table S2).

Extensive studies over the last decades have elucidated that the MTH reaction proceeds via a dual-cycle pathway mechanism governed by alkene and arene cycles.^81,82^ The slightly improved selectivity observed toward aromatics (as discussed above) in fluorinated ZSM-5 suggests a modest enhancement in the arene cycle, accompanied by a minor decrease in light olefin (C_2_–C_4_=) selectivity. For example, the C_2_–C_4_= selectivity decreases from 23.8% in parent ZSM-5 to 22.9% in F1-ZSM-5 at 2 h TOS (Figure 6c). Despite these small differences, the comparable distribution of aromatic and olefinic products indicates that the dual-cycle mechanism remains operative, with no major shift in the overall reaction pathway. Rather, the extended catalyst lifetime is more likely to be attributed to the suppression of deep methylation as evidenced by the reduced formation of C_9_ and C_10_ aromatics (Figure S20). These highly methylated aromatics are known precursors that readily undergo alkylation and cross-linking to form polyaromatic coke species, therefore mitigating polyaromatic growth and delaying deactivation.^83^ This interpretation is further supported by the reduced coke accumulation observed in the fluorinated catalysts (*vide infra*, Figure S21).

While the deactivation behavior is inherently complex and its mechanism deserves future study, the simple examination of the spent catalysts nevertheless helps to rationalize the catalytic performance of fluorinated ZSM-5 in MTH. To further visualize the deactivation behavior, photographs of the spent catalyst beds were compared (Figure S21a). The Parent ZSM-5 exhibited an intense top-coking near the methanol inlet region, indicating rapid hydrocarbon pool buildup and localized deactivation.^84^ Whereas the fluorinated samples displayed a significantly lighter coloration at the inlet, followed by a gradual darkening toward the middle of the bed. This downstream shift of the reaction front implies that the fluorine-induced modulation of acidity delays the onset of hydrocarbon-pool chemistry and distributes a more homogeneous reaction progression along the reactor.^85^ Consistent with this interpretation, Liutkova et. al.^85^ reported that acidity attenuation in Ca/ZSM-5 prolonged the initiation zone and mitigated top-coking. Complementarily, thermogravimetric analysis (TGA, Figure S21b) and differential TGA (Figure S21c) show a markedly lower coke burnoff for the fluorinated catalysts while the main oxidation temperatures remain essentially unchanged, suggesting less coke of a similar nature.^5^ For example, pristine ZSM-5 exhibited a total weight loss of ∼12.0% with a major combustion peak at 582 °C, whereas F3-ZSM-5 showed only a ∼6.3% weight loss with a comparable DTG peak temperature. The nearly identical oxidation temperature for all catalysts (Figure S21c) suggests that fluorination of ZSM-5 reduced coke amounts without altering its nature, in line with the essentially unchanged product MTH reaction pathway discussed above.

Taken together, these results indicate that fluorine does not be regarded merely as a dealumination agent, but rather as a persistent framework modifier that interacts with both Al and Si environments. Although F–Al species undergo irreversible evolution during the MTH reaction (^27^Al 3QMAS NMR, Figure S19), the stability of F–Si coordination even after high-temperature treatment (up to 800 °C) highlights the sustained presence of fluorine within the zeolite framework (Figures S22, and S23). In this context, fluorine species act cooperatively with intrinsic BASs to modulate the local acidity and reaction environment.

To further examine the impact of the transformation of F–Al species after the MTH reaction on catalytic performance and to better understand their role in the MTH process, the regenerated catalysts (denoted as Rx-ZSM-5) were re-evaluated in the MTH reaction (Figure S24). All regenerated catalysts exhibit lower methanol conversion and reduced aromatic selectivity compared to their fresh counterparts, consistent with modifications of framework Al environments under MTH conditions, as evidenced by the ^27^Al NMR results of the spent catalysts.

More importantly, R-F1-ZSM-5 shows even lower aromatic selectivity (17.2%) than R-ZSM-5 (24.0%), which correlates with the attenuation of F–Al species, implying that polarized hydroxyl species associated with F–Al environments (Figure S25) play a key role in hydrogen transfer and aromatization. This interpretation is further supported by the decreased HTI values observed for the regenerated fluorinated samples (Table S3). These findings underscore that the role of fluorine in zeolites extends beyond conventional dealumination or morphology control and should be carefully considered in the rational design of fluorine-modified zeolite catalysts. Moreover, they open new opportunities for tuning zeolite acidity, potentially enabling a broader range of catalytic applications.

## Conclusions

Fluorine has long played a crucial role in zeolite chemistry, most notably as a dealumination agent, yet its strong electronegativity and potential to alter the electronic environment of the framework have long been unclear. In this work, we systematically revisited the role of fluorine in ZSM-5 through controlled hydrothermal fluorination with NH_4_F, focusing on effects beyond conventional dealumination. The acidity of ZSM-5 was enhanced within a very mild fluorination range (NH_4_F below 300 mg g_zeolite_^–1^) through a quantitative analysis using NH_3_-TPD and pyridine FTIR. In particular, the intrinsic acidity of 275 μmol g^–1^ in parent ZSM-5 increased to 360 μmol g^–1^ upon fluorination with 300 mg g_zeolite_^–1^ NH_4_F (F3-ZSM-5). Using Al-free silicalite-1 as a reference, we demonstrate that fluorination induces the formation of Brønsted acid-like sites by polarizing the silanol. Comprehensive solid-state NMR analysis confirms that these polarized protons in silicalite-1 resonate broadly (2.5–6.0 ppm), partially overlapping with intrinsic Brønsted acid sites (BASs) in ZSM-5, yet exhibit comparatively weaker acidity (∼390 °C desorption in NH_3_-TPD). As a result, fluorination-induced mild dealumination reduced the intrinsic BAS density but simultaneously created BAS-like protons by polarization of silanol. The net result is a redistributed acid landscape rather than a simple loss of framework aluminum.

The catalytic consequences of this modified acidity were evaluated in the methanol-to-hydrocarbons reaction, where fluorinated-ZSM-5 (below 600 mg g_zeolite_^–1^) exhibited a prolonged lifetime together with enhanced aromatic selectivity. This can be rationalized by the synergetic effect between the intrinsic BASs and newly formed BAS-like protons, particularly those associated with F–Al species. The resulting reaction network suppresses deep methylation of aromatics, as evidenced by the inhibited growth from C_8_ to C_9_–C_10_ species and their subsequent evolution toward polyaromatic species.

Overall, this study establishes that fluorine functions not only as a structural but also as an electronic modifier that reshapes zeolite acidity through interactions with framework Al and Si. These findings offer a new perspective for future research on fluorine-mediated zeolite modification and synthesis, emphasizing that the influence of fluorine on the framework’s electronic structure and thus on acidity should not be overlooked, beyond the commonly attributed effects of porosity adjustment or dealumination.

## Supplementary Material



## Notes

Experimental section and characterization details; definition of the catalytic results; SEM images; calculated relative aluminum ratio; BET surface area; TEM images; NH_3_-TPD profiles of silicalite-1, and fluorinated silicalite-1; the pyridine adsorbed FTIR spectra; NH_3_-TPD profiles and catalytic behavior test of silicalite-1, and fluorinated silicalite-1; ^1^H MAS NMR spectra of silicalite-1, and fluorinated silicalite-1; selectivity of xylene, C_9_, and C_10_ aromatics of parent ZSM-5 and F-ZSM-5 catalysts; reacted fixed-bed catalysts and TGA analysis and ^27^Al 3QMAS NMR analysis on spent catalysts; and regeneration of spent catalysts and further MTH catalytic behavior evaluation (PDF)
