# Supplementary material for: Beyond Dealumination: Does Fluorine Reshape Zeolitic Acidity?
Source: J Am Chem Soc. 2026 Jun 11;148(29):30905–19. doi: 10.1021/jacs.6c04557 (PMC13426252; doi:10.1021/jacs.6c04557)
Supplement: Supplementary file 1 [file ja6c04557_si_001.pdf]

Beyond Dealumination: Does Fluorine Reshape Zeolitic Acidity?

*Lu Song[1], Natalia Morlanés[1], Edy Abou–Hamad[2], Javier Ruiz-Martínez[1]**

[1]King Abdullah University of Science and Technology, KAUST Catalysis Center (KCC), Thuwal 23955, Saudi Arabia

[2]King Abdullah University of Science and Technology, Core lab, solid-state NMR, Thuwal 23955, Saudi Arabia.

*e-mail: javier.ruizmartinez@kaust.edu.sa

Experiments

Characterization. Inductively coupled plasma optical emission spectroscopy (ICP-OES) was executed using an Agilent 5110 ICP-OES and Ultra WAVE Microwave Digestion System (Milestone). The acid digestion of the sample was carried out using a mixture of hydrochloric acid, nitric acid, and hydrofluoric acid (6:2:1 v/v/v ratio) at 270 °C and 35 bar.

Powder X-ray diffraction (PXRD) measurements were carried out on a Bruker D8 diffractometer using Cu Kα radiation (1.5 Å), operated at 40 kV and 40 mA. The data were collected in a 2θ range from 0 to 90° with a step width of 0.03° and a scan speed of 3.0 s $step^{-1}$.

Nitrogen (N_2) physisorption was conducted at −196 °C using a Micromeritics ASAP 2420 high-throughput analysis system. Before the measurement, all samples were degassed at 300 °C under a vacuum for 12 h. The micropore volumes were estimated using the Dubinin−Radushkevich method.

Scanning electron microscopy (SEM) measurements were conducted from a Quattro field emission scanning electron microscope (operated at an acceleration voltage of 10 keV) at approximately a 10 mm working distance. Transmission electron microscopy (TEM) measurements were carried out using Tecnai Twin microscopy at an acceleration voltage of 200 kV, and the samples diluted in ethanol were cast onto a copper grid and subsequently dried at 60 °C for 30 min.

Temperature-programmed desorption of ammonia (NH_3-TPD) measurements were carried out on a Microactivity Reference setup (PID Eng &Tech). The inlet and outlet NH_3 concentrations were continuously monitored with a MultIGasTM 2030 FTIR gas analyzer. 100 mg of catalyst was located in a fixed-bed quartz reactor. Prior to analysis, the catalyst was pretreated in a flow of N_2 at 400 °C for 40 min with a ramping rate of 10 °C min^{-1}. After cooling to 100 °C, the catalyst was exposed to 3% NH_3 (balanced in 46 mL min^{-1} N_2) for 40 min to achieve complete adsorption. Subsequently, the physiosorbed NH_3 was removed by purging with 200 mL min^{-1} of a N_2 flow for 80 min. The desorption step was then performed by heating the sample from 100 to 700 °C at a ramp rate of 10 °C min^{-1} under a continuous N_2 flow.

Fourier transform infrared (FTIR) spectroscopy using pyridine as a probe molecule was conducted on a Nicolet 6700 spectrometer fitted with a mercury cadmium telluride (MCT-B) detector possessing a low-frequency cutoff. Samples were first dehydrated at 450 °C for 12 h under vacuum with a ramp rate of 5 °C min^{-1}. Pyridine was then introduced at its vapor pressure under

ambient conditions and allowed to saturate the catalyst for 30 min. Afterwards, weakly adsorbed physiosorbed pyridine was removed by evacuating the catalysts at 150 °C for 2 h. FTIR spectra were recorded over the 1000–5000 cm^{-1} region at a 4 cm^{-1} resolution, averaging 64 scans for each sample. The acidity was defined as D

$$D = \frac{A \times R^2}{E \times m}$$

where R is the radius of catalyst pellets (cm), A is the absorbance area corresponding to BASs or Lewis acid sites (LASs) (cm^{-1}), E is the extinction coefficient (1.67 $cm \cdot \mu mol^{-1}$ for BASs and 2.22 $cm \cdot \mu mol^{-1}$ for LASs)[20], and m is the mass of the catalyst (mg).

Solid-state ^{27}Al and ^{29}Si MAS NMR experiments were performed on a Bruker 900 MHz with a 3.2 mm HXY probe. ^{19}F and ^{1}H MAS NMR spectra were recorded on a Bruker 600 MHz with a 2.5 mm FXY probe (25 kHz) and 3.2 mm HXY probe (MAS frequency 20 kHz), respectively. For the ^{27}Al single pulse experiment, π/6 pulses were applied at a MAS frequency of 20 kHz at room temperature using a recycled delay of 0.5 s with an accumulation of 1024 scans. The 2D ^{27}Al triple-quantum MAS (3Q MAS) spectra were recorded using a 4.4 μs excitation pulse, 1.4 μs conversion pulse at the 178 kHz radio frequency field, and 20 μs selective pulse at the 12.5 kHz field with the accumulation of 4800 scans. The ^{29}Si MAS NMR experiments and ^{1}H–^{29}Si MAS NMR experiments (both 1D and 2D) were conducted with a MAS frequency of 20 kHz at room temperature. Single ^{29}Si MAS NMR spectra were recorded using a recycled delay of 20.0 s with an accumulation of 1024 scans. The 1D ^{1}H–^{29}Si cross-polarization (CP) MAS NMR experiments with a recycled delay of 1.0 s, 44 ms acquisition time, and 10240 scans. The 2D ^{1}H–^{29}Si Lee–Goldberg HETCOR spectra were recorded using a 1 s recycle delay with an accumulation of 10240 scans. ^{1}H and ^{29}Si chemical shifts were referenced externally to adamantane and hexamethylcyclosiloxane, respectively.

^1H MAS NMR spectra were performed with a recycle delay of 4 s, 2.500 μs pulse duration, and 32 scans. 2D ^1H double-quantum single-quantum (DQ–SQ) MAS NMR experiments were performed using a back-to-back recoupling sequence with double-quantum coherences with a duration of 120 μs. Direct ^{19}F MAS NMR spectra were recorded with a 10 s recycled delay, 3.5 μs pulse duration, and an accumulation of 10240 scans. The 2D $^{19}F-^{19}F$ Nuclear Overhauser Effect Spectroscopy (NOESY) experiments were conducted with a 2 s recycled delay, 0.05 s mixing time, and accumulations of 128 scans.

Thermal gravimetric analysis coupled with mass spectrometry (TGA−MS) was performed using a Mettler Toledo TGA/ DSC1 Stare System under airflow. The spent catalysts (∼10 mg) were tested in the range from 50 to 800 °C with a ramping rate of 5 °C min^{-1} under continuous airflow of 50 mL min^{-1}.

Catalytic Activity Test. Catalytic tests were performed on a four-channel Flowrence XD from Avantium. 25 mg of catalysts were mixed uniformly with 300 μL of SiC before loading to avoid hot spots. The reactors were 300 mm long quartz tubes inserted in a furnace. The outside diameter and inside diameter of the tubes were 3 and 2 mm, respectively. Before feeding the reaction mixture, all samples were pretreated with a N_2 atmosphere for 2 h at 400 °C. Then, the catalytic activity of the catalyst was tested at 400 °C. N_2 was used as carrier gas, keeping a molar MeOH: N_2 ratio with a weight hourly space velocity (WHSV) of 16 g of MeOH $g_{zeolite}^{-1}$ h^{-1} at 1 bar. The liquid feed was evenly distributed to the four reactors using a one-glass microfluidic distributor chip. The criteria and definitions used for evaluating the catalytic results are summarized in the supporting information. The unreacted methanol and reaction products were analyzed by a gas chromatographer (Agilent 7890B) with three detectors: one thermal conductivity detector (TCD) and two flame-ionized detectors (FID). The TCD channel has a PPQ as blackflush column, a

Hayesep Q column for the separation of CO_2, and a Molsieve as an analytical column for the separation of He, H_2, N_2, CH_4, and CO. All other compounds (water, hydrocarbons, and oxygenates) are backflushed. The FID has a 10 m precolumn with a wax stationary phase. The separation of C_1-C_5 hydrocarbons is carried out on a 30 m Gaspro stationary phase. Separation of methanol and aromatics is carried out on a 30 m wax stationary phase. Methanol and dimethylether (DME) were considered as the reactants with the conversion defined as,

$$X = \left[1 - \frac{C_{\text{eff}}}{C_{\text{feed}}}\right] \cdot 100 \qquad \text{Equation 1}$$

where C_{eff} is the concentration of both methanol and DME in the effluent and C_{feed} is the concentration of methanol in the feed. The selectivity S_i of the product i is defined as,

$$S_i = \left[\frac{C_i}{C_{\text{t,eff}}}\right] \cdot 100 \qquad \text{Equation 2}$$

where C_i is the concentration of product i, in the effluent and $C_{t,eff}$ is the total concentration of product in the effluent. All concentrations used in the calculations were expressed on a carbon basis by weigthing each compound by its corresponding carbon number.

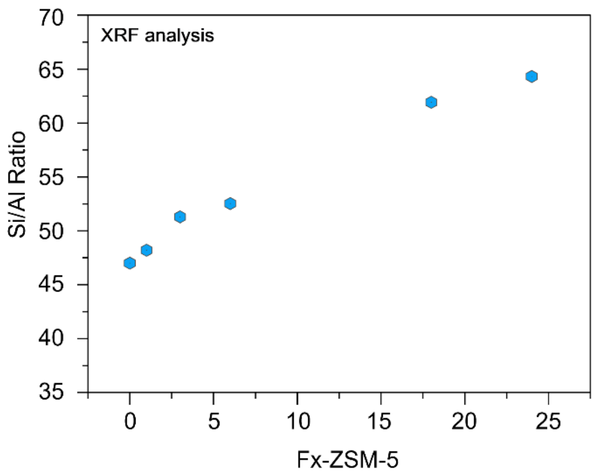

Figure S1. Compositional analysis of parent ZSM-5 and fluorinated ZSM-5 as a function of fluorination level (x = number of 100 mg NH_4F portions added in the hydrothermal process). X-ray fluorescence (XRF) analysis results. It should be noted that the absolute Si/Al ratios obtained from different techniques may vary due to differences in measurement principles; however, a consistent increasing trend of Si/Al ratio with fluorination level is observed.

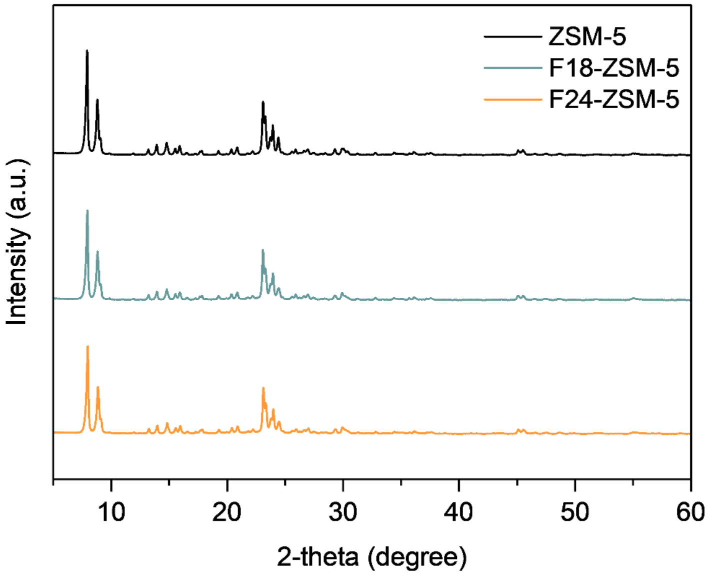

Figure S2.XRD of parent ZSM-5 and ZSM-5 with high NH_4F content (1800 and 2400 mg per gram of zeolites).

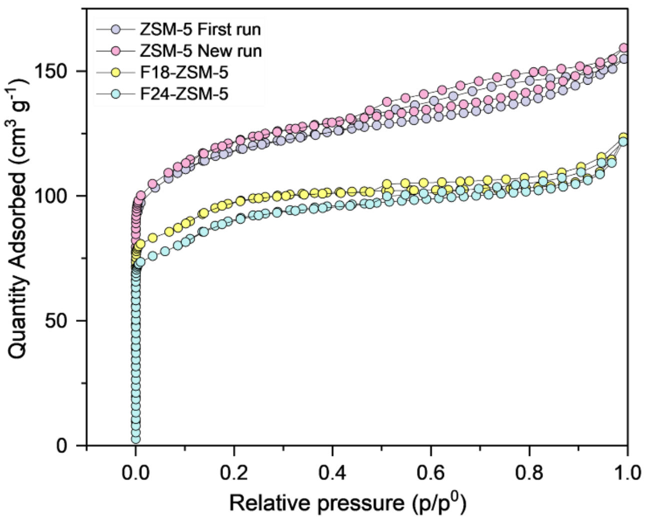

Figure S3. N_2-physissoprtion isotherms of parent ZSM-5 and ZSM-5 with high NH_4F content (1800 and 2400 mg per gram of zeolites).The repeated measurement of the parent ZSM-5 (denoted as *ZSM-5 New run*) is included as a reference, showing excellent agreement with the initial measurement (ZSM-5 First run) and confirming the reliability and reproducibility of the experimental procedure.

No obvious mesopore formation is observed even at high NH_4F loadings, as evidenced by the N_2 physisorption results. This behavior suggests that, under the present conditions, fluorination does not lead to effective mesopore generation but instead induces framework degradation and structural rearrangement (as supported by further discussion).

This behavior is consistent with previous reports that mesopore formation in zeolites during NH_4F etching is governed by stochastic hydrolysis and removal of framework atoms rather than a controlled or directed process.[1] This indicates that mesopore formation is not an inherent or inevitable outcome of NH_4F treatment, Nevertheless, the fluorination clearly weakens the framework stability, as further supported by the subsequent structural and catalytic analyses.

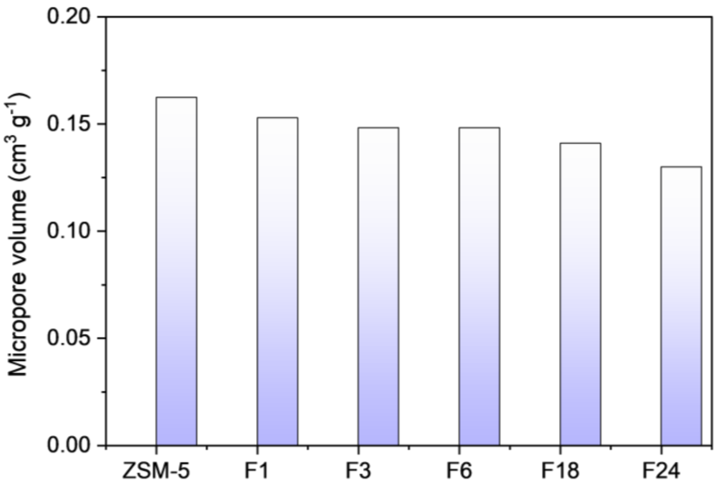

Figure S4. Micropore volume of the parent ZSM-5 and fluorinated ZSM-5 samples derived from N_2 physisorption isotherms using the Dubinin–Radushkevich (D–R) method.[2,3]

As reported by Qin et al., the NH_4F etching process involves stochastic hydrolysis and removal of framework atoms, with dissolution ranging from partial framework removal to extensive degradation of the crystal outer regions.[1] Therefore, the loss of microporosity observed here is consistent with framework degradation rather than the formation of well-defined mesoporosity. In addition, a fraction of fluorine species may reside within the micropores and form local coordination environments, which could partially reduce the accessibility of pore space. However, this effect is expected to be secondary and cannot account for the overall decrease in micropore volume, which is primarily attributed to framework dissolution and structural rearrangement (as further supported by the framework analysis including the ^{27}Al NMR and ^{19}F NMR before and after reaction, *vide infra*).

Sample	*Micropore volume* *cm^3 g^{-1}*	*Mesopore volume* *cm^3 g^{-1}*
ZSM-5	0.162	0.089
F1-ZSM-5	0.152	0.099
F3-ZSM-5	0.154	0.105
F6-ZSM-5	0.148	0.103
F18-ZSM-5	0.130	0.071

F24-ZSM-5	0.119	0.065

Table S1. Textural properties of parent ZSM-5 and fluorinated ZSM-5 derived from N_2 isotherms. The micropore volume decreases progressively with increasing NH_4F loading (from 0.162 to 0.119 cm^3 g^{-1}). Instead, the mesopore volume exhibits a non-monotonic evolution, increasing from 0.089 cm^3 g^{-1} for the parent ZSM-5 to a maximum of 0.105 cm^3 g^{-1} for F3-ZSM-5, followed by a significant decrease at higher fluorination levels (0.065 cm^3 g^{-1} for F24-ZSM-5).

This behavior reflects two distinct regimes. At low fluorination levels (below 600 mg g^{-1} zeolite), the decrease in micropore volume is partially compensated by the formation of mesopores due to controlled framework etching, where partial dissolution generates additional mesoporosity. In contrast, at higher fluorine loadings (> 600 mg g^{-1} zeolite), both micropore and mesopore volumes decrease, indicating excessive framework degradation and pore collapse rather than pore transformation.

Consistently, the N_2 adsorption–desorption isotherms show that the hysteresis loop becomes significantly less pronounced at higher fluorine loadings (F18 and F24-ZSM-5). Since hysteresis arises from capillary condensation in mesopores, this behavior suggests that excessive fluorination leads to pore collapse or disruption of the pore network. This observation is supported by SEM images (below Figure S5), which reveal pronounced surface dissolution and the blurring of intergranular boundaries, suggesting progressive structural degradation. Therefore, the combined evolution of micropore volume and mesopore volume demonstrates that the structural transformation proceeds from controlled framework etching to partial collapse as the fluorine content increases.

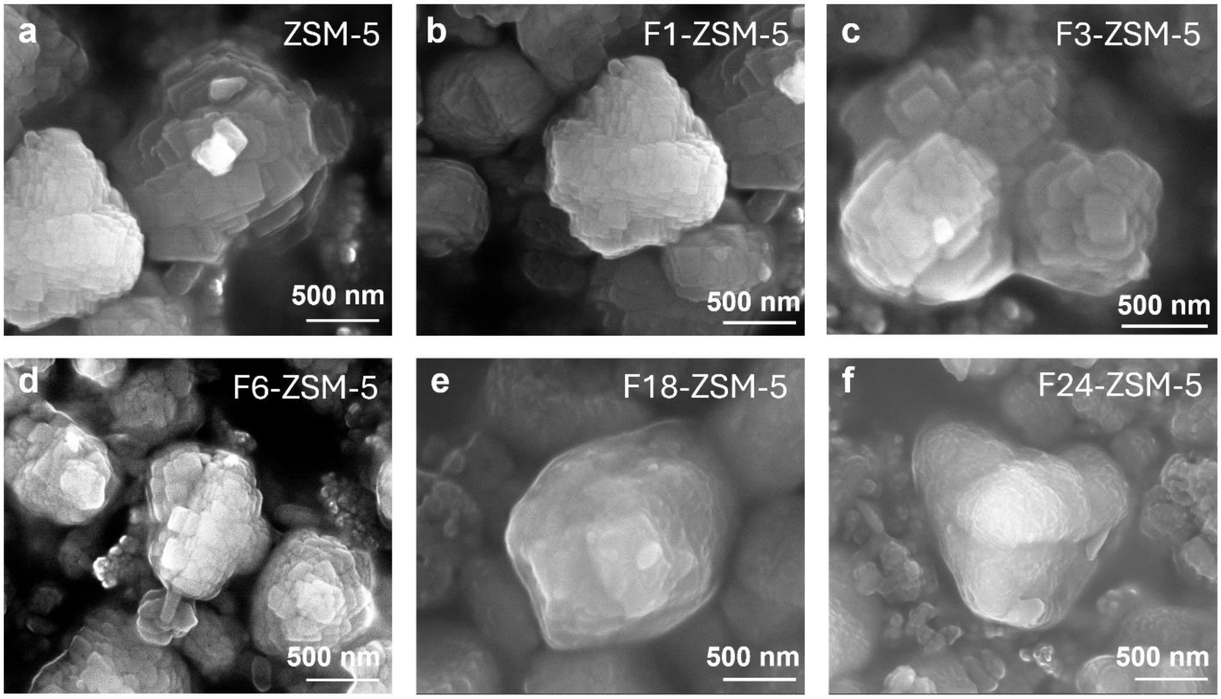

Figure S5. Scanning Electron Microscopy (SEM) images of the (a) parent ZSM-5, (b) F1-ZSM-5, (c) F3-ZSM-5, (d) F6-ZSM-5, (e) F18-ZSM-5, and (f) F24-ZSM-5. The F1-ZSM-5 sample retains a similarly well-defined morphology, with smooth surfaces and sharp crystal edges, indicating that the overall structure is largely preserved at low fluorine loading. However, starting from F3-ZSM-5, noticeable surface modifications begin to appear. The crystal surfaces become less well-defined, and subtle roughening is observed, suggesting the onset of structural perturbation induced by fluorination. At intermediate fluorination levels (F6-ZSM-5), these changes become more pronounced. At higher fluorine contents (F18 and F24), the morphological changes become significantly more severe. The crystals appear increasingly rounded, accompanied by a clear loss of faceting and structural definition. These features indicate substantial structural degradation, rather than simple surface etching. Overall, the SEM observations reveal a progressive evolution from well-defined crystalline morphology to severely perturbed structures with increasing fluorination, with the most pronounced degradation observed for F18 and F24 samples.

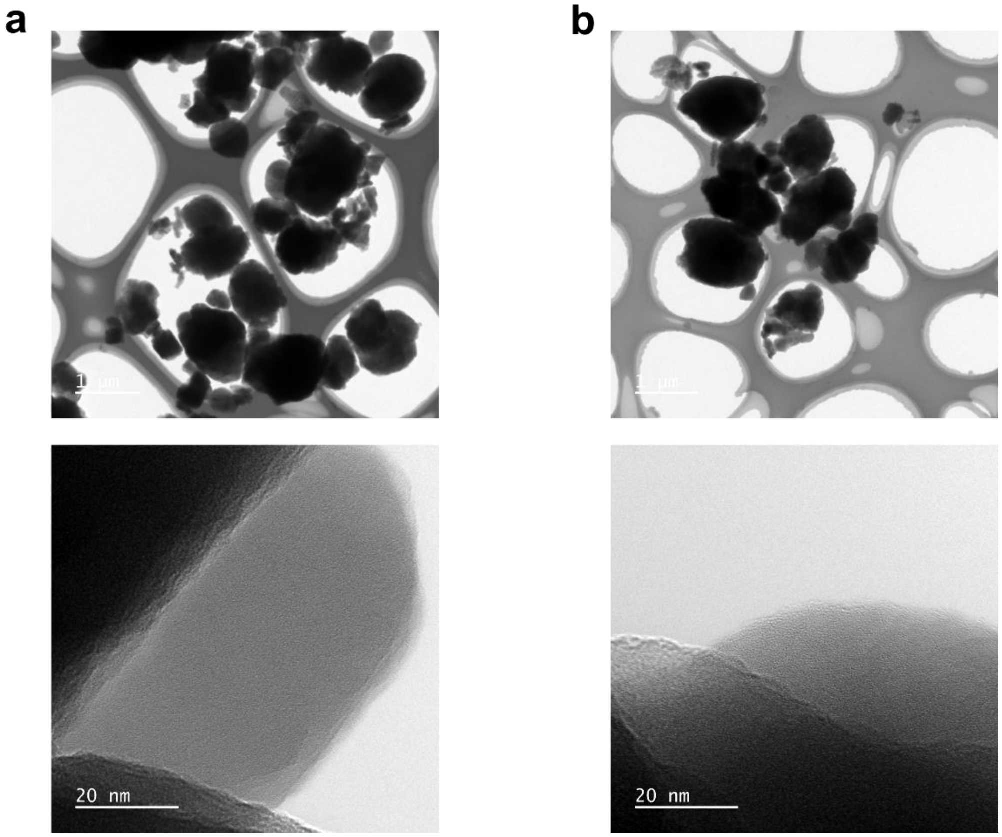

Figure S6. Transmission electron microscopy (TEM) (top) and high-resolution TEM images (bottom) of (a) parent ZSM-5 and (b) F3-ZSM-5 catalyst, showing well-preserved crystal morphology and framework integrity after fluorination.

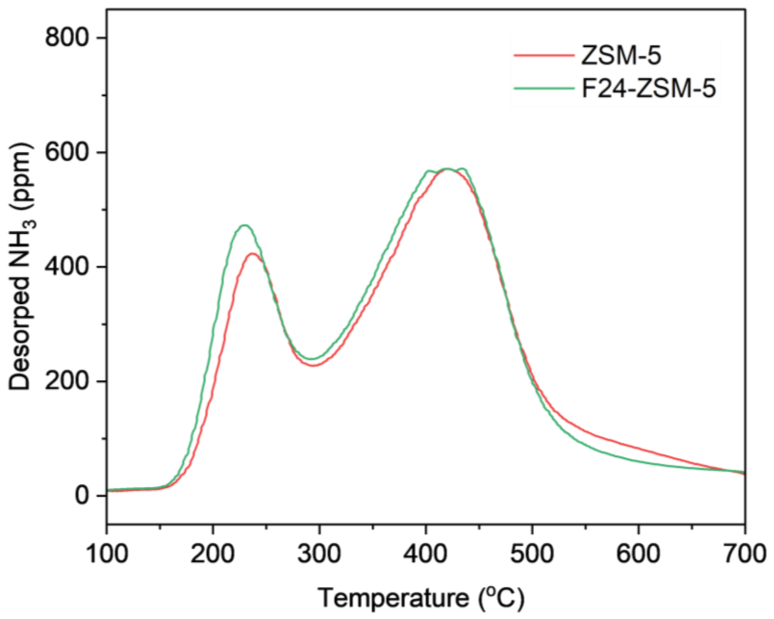

Figure S7. NH_3 temperature-programmed desorption (NH_3-TPD) profiles of pristine ZSM-5 and F24-ZSM-5 catalyst. Even at the highest fluorination level (F24-ZSM-5), with ~30% loss of Al in ZSM-5, the NH_3-TPD signal remains comparable to that of pristine ZSM-5, confirming that the overall acidity is maintained through fluorine-induced acidity formation.

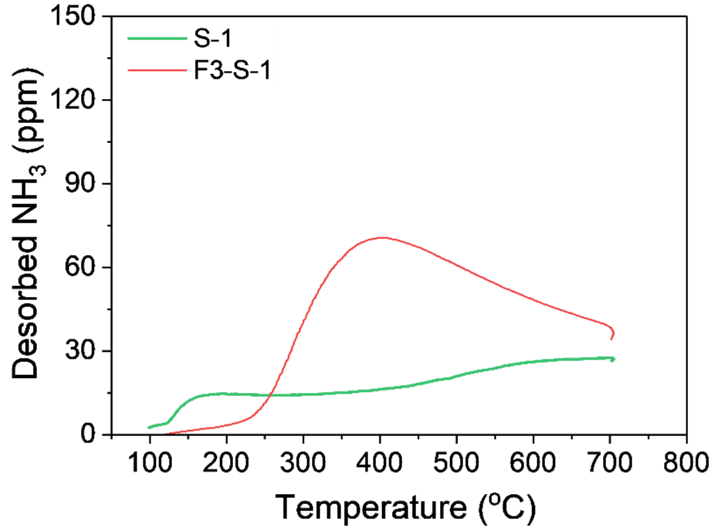

Figure S8. NH_3 temperature-programmed desorption (NH_3-TPD) profiles of pristine silicalite-1 and fluorinated silicalite-1 (F3-S-1).

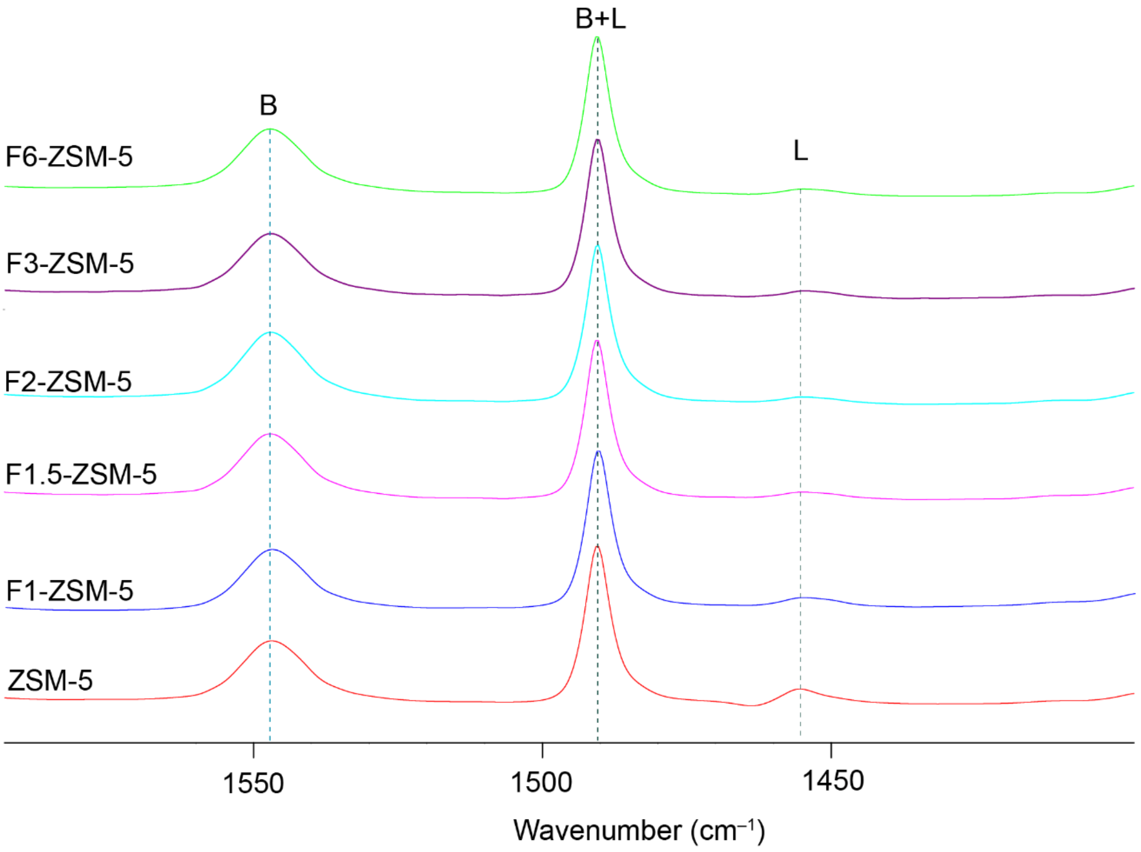

Figure S9. Pyridine-adsorbed Fourier Transform Infrared (FTIR) spectra of the parent and fluorinated ZSM-5 samples. The spectra were normalized for clarity. Quantitative acid-site concentrations were determined from the integrated band areas normalized by pellet mass, using the bands at ~1545 cm^{-1} (Brønsted) and ~1455 cm^{-1} (Lewis) using IMEC values of 1.67 and 2.22 cm μmol^{-1}, respectively.[4]

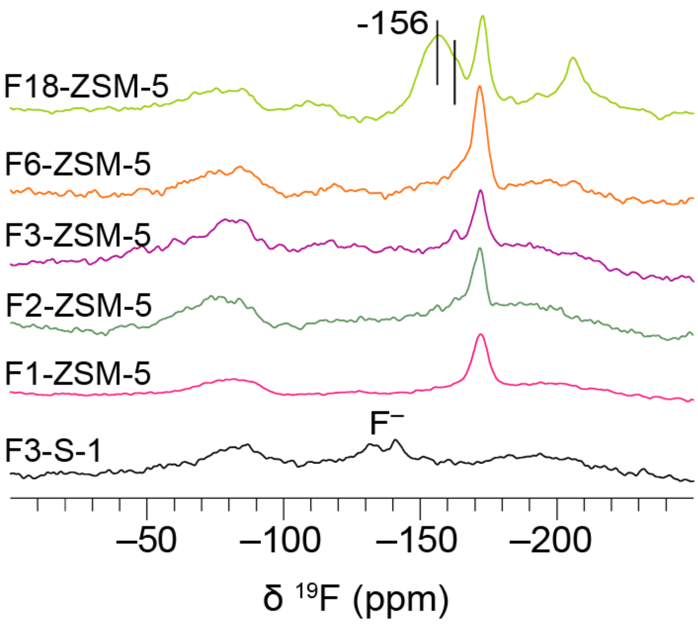

Figure S10. ^{19}F MAS NMR spectra of F-ZSM-5 and F-silicalite-1. An additional resonance at −156 ppm is observed only for highly fluorinated sample (F18-ZSM-5, treated with NH_4F 1800 mg $g^{-1}_{zeolites}$), which is included here mainly as an extreme reference point for comparison with the other samples. At such an excessive fluorination level, the severe framework deterioration (as discussed in XRD results in Figure 1) goes beyond the scope of the present study. This signal is tentatively attributed to fluorine accumulated in highly defective or Si-rich regions (e.g., F-rich disordered domains), rather than to Al-coordinated fluorine species. Importantly, despite this extreme case, the overall ^{19}F MAS NMR spectra across the series unambiguously confirms the successful incorporation of fluorine and its increasing abundance with increasing NH_4F content.

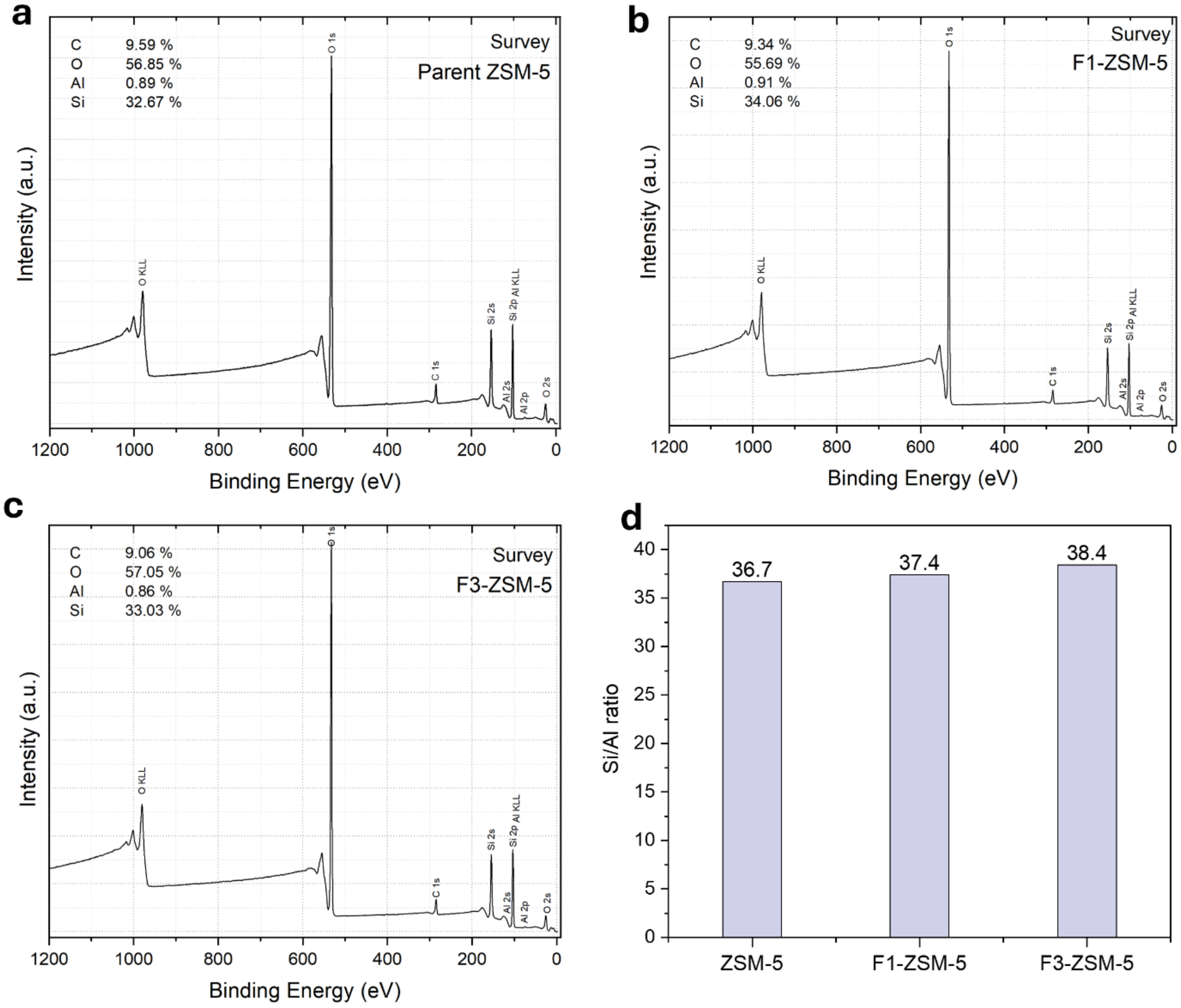

Figure S11. A surface-sensitive technique used to analyze elemental composition and chemical states based on the binding energy of photoemitted electrons. XPS survey spectra of the parent ZSM-5 (a), F1-ZSM-5 (b), and F3-ZSM-5 (c). (d) Surface Si/Al ratios obtained from XPS, showing a gradual increase after NH_4F treatment.

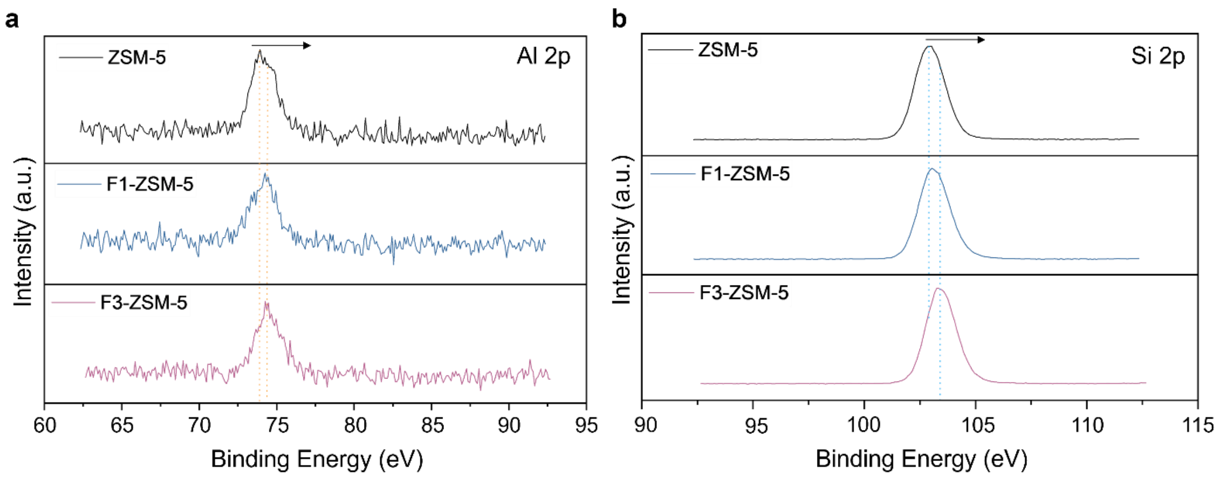

Figure S12. High-resolution XPS spectra of Al 2p and Si 2p for ZSM-5 and fluorinated samples (F1 and F3). All binding energies were calibrated using the C 1s peak at 284.8 eV as a reference.[5–8]

High-resolution XPS spectra show that the Al 2p binding energy shifts slightly from 73.9 eV for parent ZSM-5 to 74.3 and 74.5 eV for F1-ZSM-5 and F3-ZSM-5, respectively. while the Si 2p peak shifts from 102.9 eV to 103.1, and 103.4 eV for F1-ZSM-5 and F3-ZSM-5, respectively, after fluorination. These changes indicate a modification of the local electronic environment of both Al and Si species upon NH_4F treatment. Combined with the ^{19}F MAS NMR results, which confirm the presence of F–Al and F–Si environments, these data provide strong evidence that fluorine is incorporated into the zeolite framework and induces electronic perturbation of the local structure.

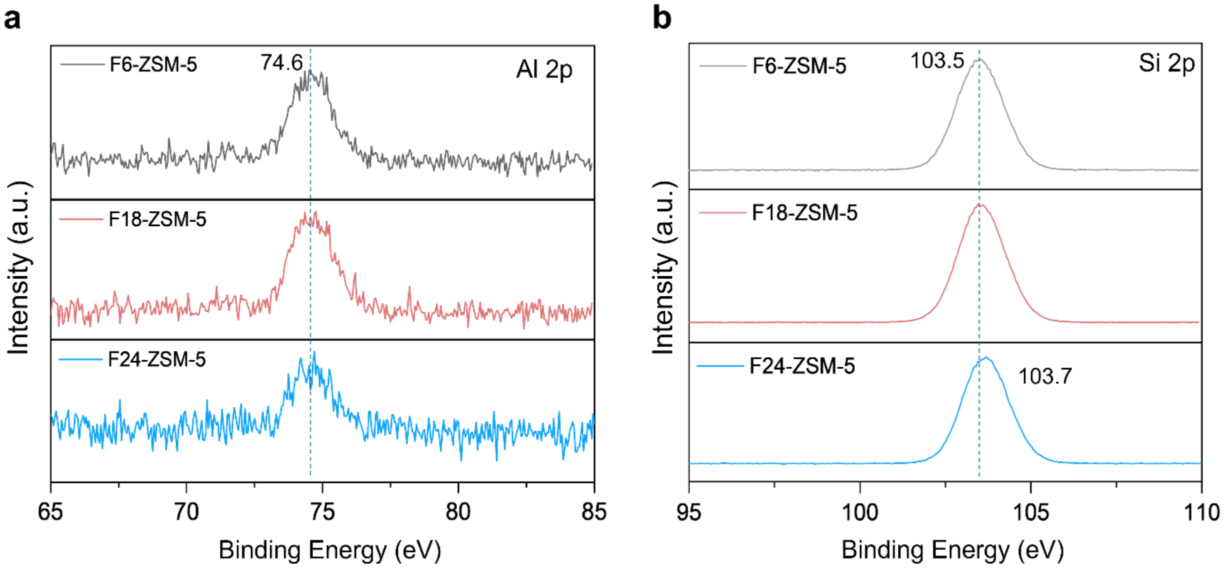

Figure S13. High-resolution XPS spectra of Al 2p and Si 2p for ZSM-5 and fluorinated samples (F6, F18, and F24-ZSM-5).

For highly fluorinated samples (F6 to F24), the Si 2p peak remains around 103.5 to 103.7 eV, while the Al 2p signal becomes broadened and less well resolved, especially for F24-ZSM-5. This broadening is consistent with a more heterogeneous Al environment and progressive dealumination under high NH_4F loading.

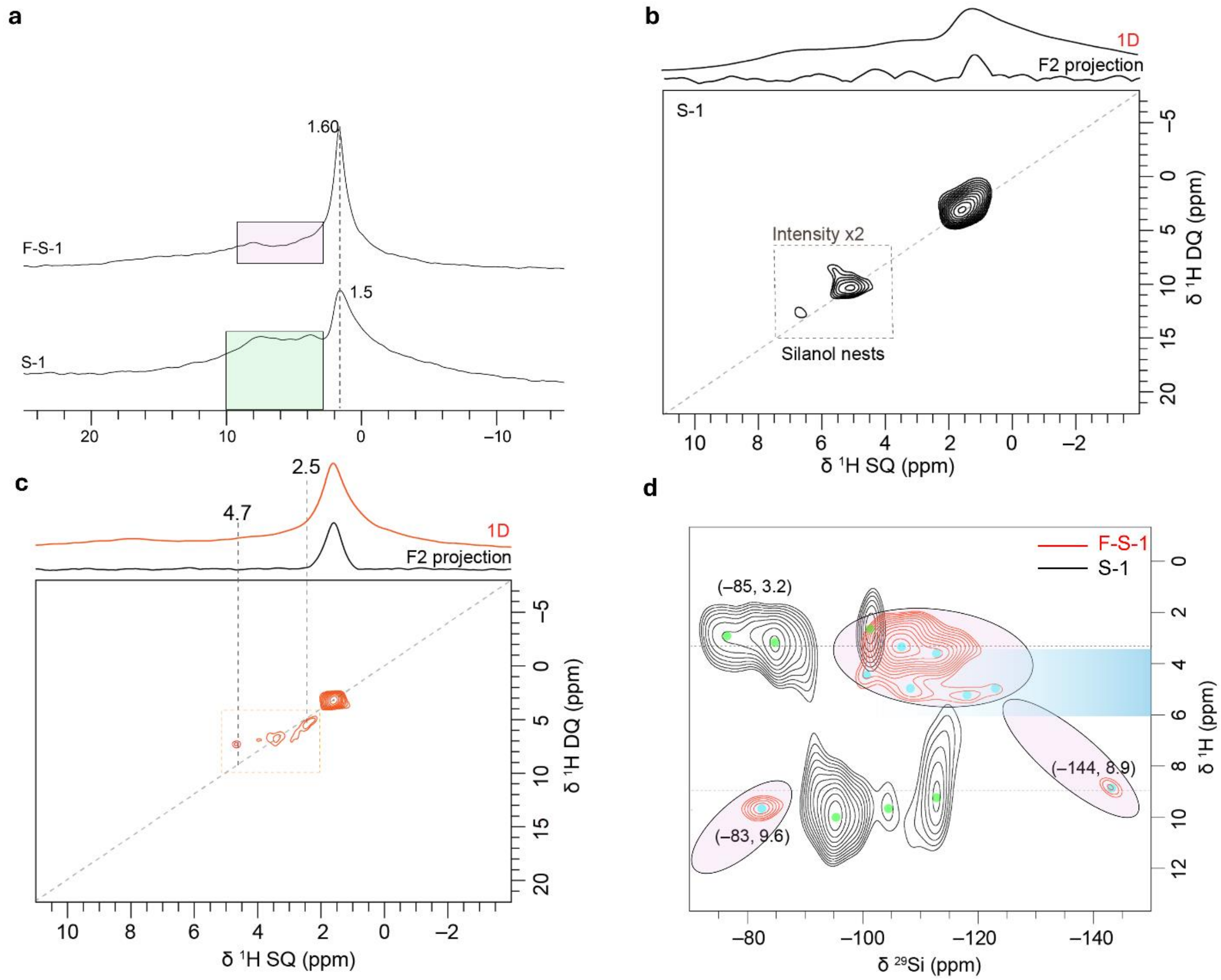

Figure S14. Solid-state NMR analysis of proton environments in silicalite-1 and F-S-1 catalysts. Direct ^{1}H MAS NMR spectra of the pristine silicalite-1, and F3-S-1. (a) ^{1}H NMR spectra of parent and fluorinated S-1. 2D ^{1}H DQ–SQ MAS NMR results of (b) S-1, and (c) F-S-1. Since the defects healing and the limited information given by the 2D^{1}H DQ–SQ NMR, we further applied experiments on controlled dehydrate samples at a high-vacuum line at 450 °C for 12 h at ~10^{-6} bar.

Notably, silanol species at chemical shifts below ~2.5 ppm is not clearly observed in the ^{1}H–^{29}Si HETCOR spectra, which can be attributed to weak dipolar coupling and enhanced proton mobility, which can significantly reduce the effective dipolar coupling to nearby Q_3 sites. In particular, molecular motions (eg. local reorientation or isotropic tumbling) can lead to partial averaging of dipolar interactions, which significantly reduces cross-polarization efficiency.[9–11] In addition, the corresponding signals are not prominent in the ^{1}H–^{1}H DQ (e.g., DQ-BABA) spectra, suggesting

that these proton species are not strongly dipolar-coupled to neighboring protons and are likely spatially isolated. However, clear correlations are observed in the ^{1}H–^{29}Si HETCOR spectra for newly emerged proton environments after fluorination. This indicates that, although these protons are not involved in strong proton–proton interactions, they remain sufficiently coupled to nearby silicon sites. Such behavior is consistent with isolated but electronically perturbed proton species, likely associated with fluorine-modified framework environments.

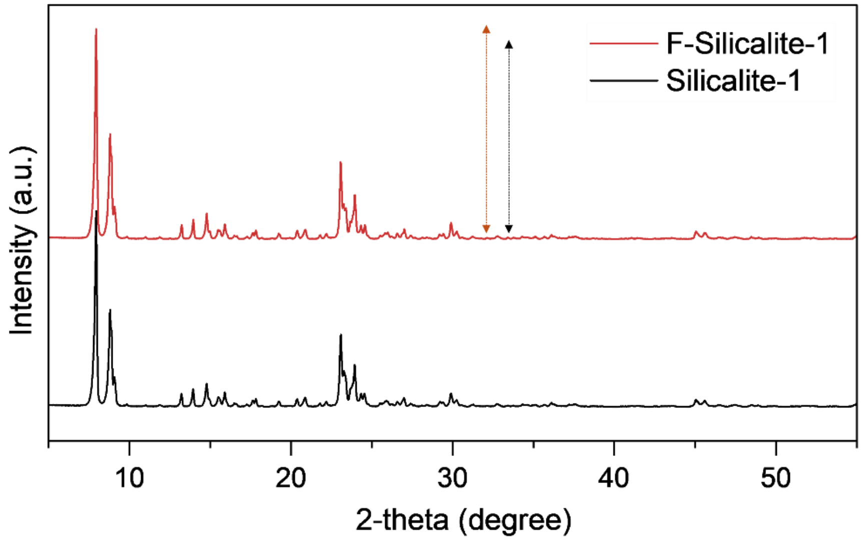

Figure S15. XRD patterns of silicalite-1 (S-1) and fluorinated silicalite-1 (F-S-1). A change in the difference in the relative peak intensities is observed after fluorination. This trend, importantly, in combination with the ^{29}Si NMR data and ^{1}H NMR data discussed in the main text (Figure 4), the increased Q^4 species in the ^{29}Si spectra after fluorination accompanied with the decreased silanol defects in ^{1}H NMR results, provides consistent evidence of structural reorganization upon fluorination, suggesting that the fluorine not only interacts with framework Si but also contributes to the healing of defects in silicalite-1.[12,13]

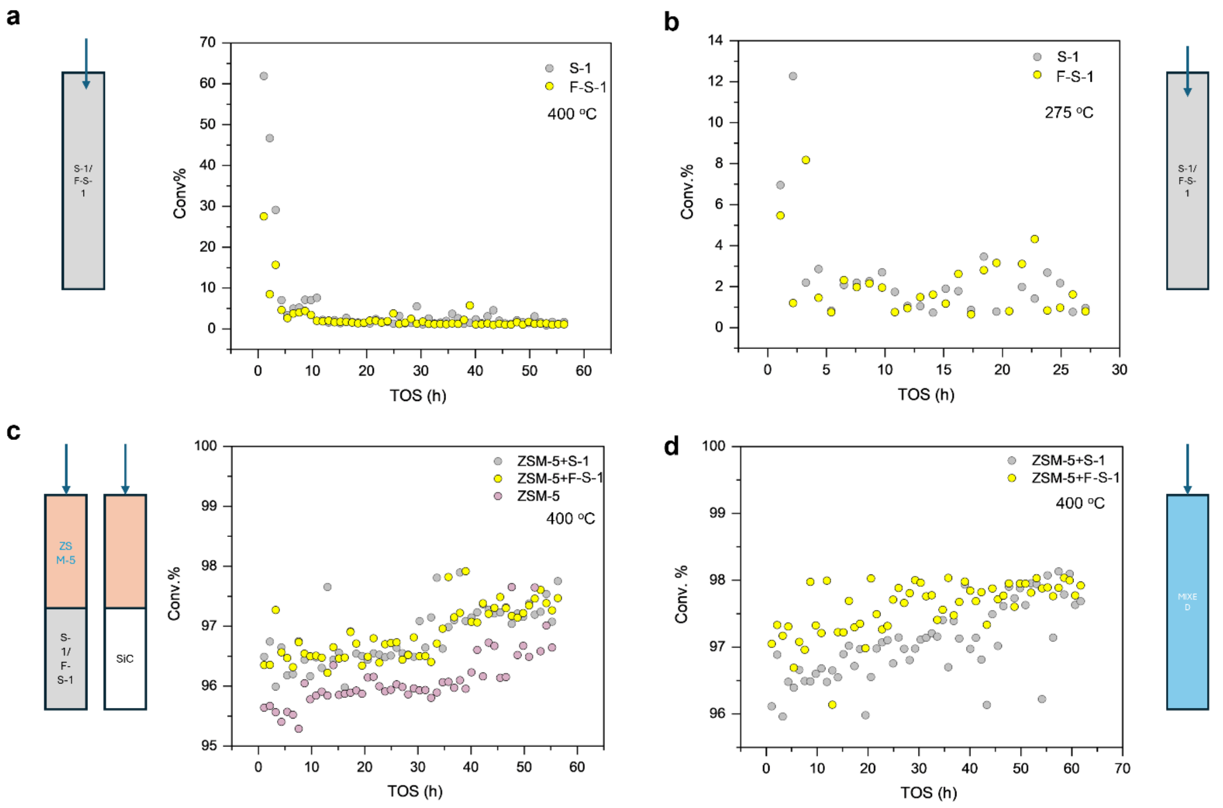

Figure S16. Catalytic performance of parent silicalite-1 (S-1) and fluorinated silicalite-1 (F-S-1) in the methanol-to-hydrocarbon (MTH) reaction. MTH reaction over S-1 and F-S-1 at (a) 400 °C and (b) 275 °C. The potential synergistic effect with parent ZSM-5: (c) two-bed reactor configuration with ZSM-5 placed in the upper bed and S-1 (or F-S-1) in the lower bed, and (d) physical mixture of catalysts. WHSV = 2.4 h^{-1}

To evaluate the intrinsic catalytic behavior of pure-silica materials, methanol-to-hydrocarbon (MTH) reactions were first performed over silicalite-1 (S-1) and fluorinated silicalite-1 (F-S-1) at 400 °C and 275 °C (WHSV = 2.4 h^{-1}), as shown in Figures S19a and S19b, respectively. At both temperatures, very low methanol conversion was observed for S-1 and F-S-1, with comparable conversion levels between the two samples. This indicates that neither material is able to sustain the hydrocarbon pool mechanism required for the MTH reaction, despite the presence of a small number of weak acid sites in F-S-1.

To further explore potential synergistic effects, these materials were combined with ZSM-5 using both two-bed reactor configurations and physical mixtures. In the two-bed configuration (Figure S19c), ZSM-5 was placed in the upper bed, while S-1 or F-S-1 was placed in the lower bed, denoted as ZSM-5/S-1-TB and ZSM-5/F-S-1-TB, respectively. Compared to ZSM-5 alone (conversion of 95.5%), a slightly higher methanol conversion (~96.5%) was observed for both ZSM-5/S-1-TB and ZSM-5/F-S-1-TB. However, given the small difference (~1%), this suggests a possible but at most limited contribution from the downstream bed.

While in the case of physical mixtures (PM) (Figure S19d), a slightly higher methanol conversion (~96.5%) is observed for the ZSM-5/F-S-1 (ZSM-5/F-S-1-PM) system compared to the ZSM-5/S-1 combination (ZSM-5/S-1-PM). However, the difference is also relatively small (~1%) and is not considered statistically meaningful, indicating only a limited impact of fluorination within the pure silica component is limited under these conditions.

Besides, the product selectivity profiles remain largely similar across different catalyst configurations (two-bed and physical mixture) (Figure S20), indicating that the overall reaction network is not altered. The similar distribution of major products, including C2–C4 olefins, C2–C4 paraffins, C5–C7 hydrocarbons, and aromatics, shows comparable trends with time-on-stream.

These results suggest that the fluorine-induced polarized hydroxyl species in F-S-1 generate weak acid sites that are insufficient to independently drive the MTH reaction but rather act cooperatively. This can be attributed to both their weak acidity and limited concentration. However, these species contribute when coexisting with the Brønsted acid sites (BAS) in ZSM-5 frameworks, enabling the pronounced changes in catalytic behavior in both catalyst lifetime (prolong the lifetime) and product distribution (enhanced aromatic selectivity). Such cooperative effects likely require spatial proximity between the newly generated acidic species and the classical BASs in ZSM-5, enabling their interaction within the hydrocarbon pool mechanism.

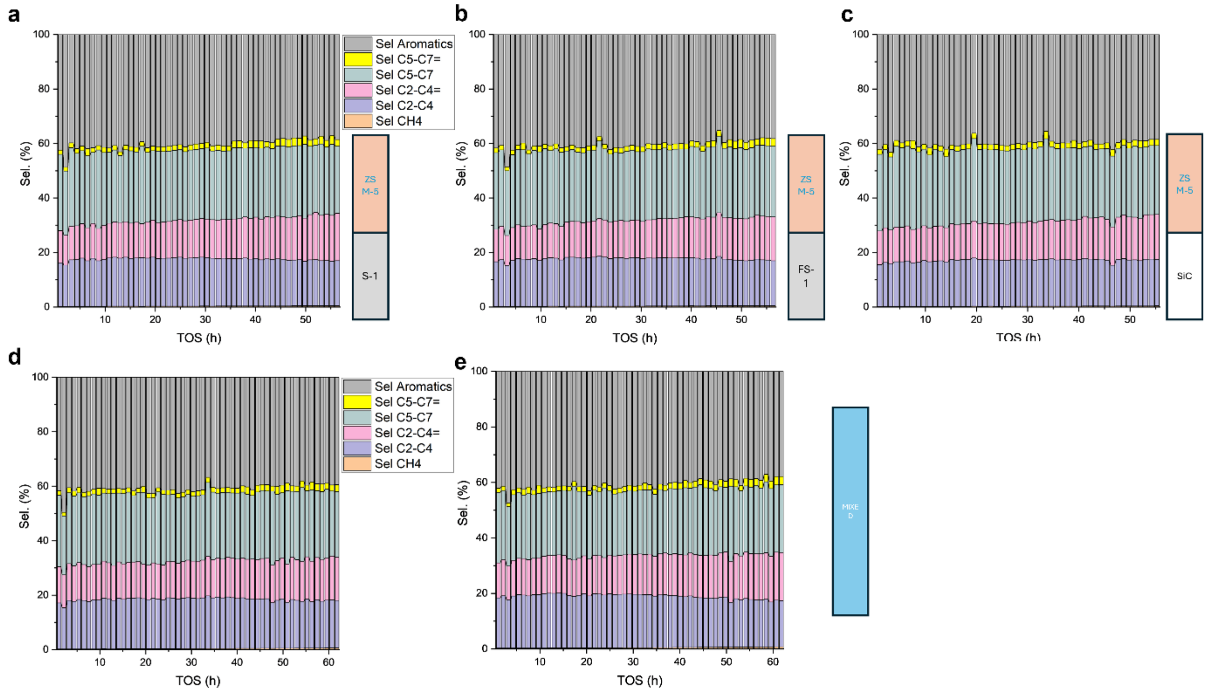

Figure S17. Product distribution as a function of TOS for different catalyst configurations. Two bed (TB) catalysts: (a) parent ZSM-5/S-1-TB, (b) ZSM-5/ZSM-5-TB, and (c) ZSM-5/SiC-TB, physical mixture (PM) of catalysts (d) ZSM-5/S-1-PM, and (e) ZSM-5/F-S-1-PM. WHSV = 2.4 h^{-1}

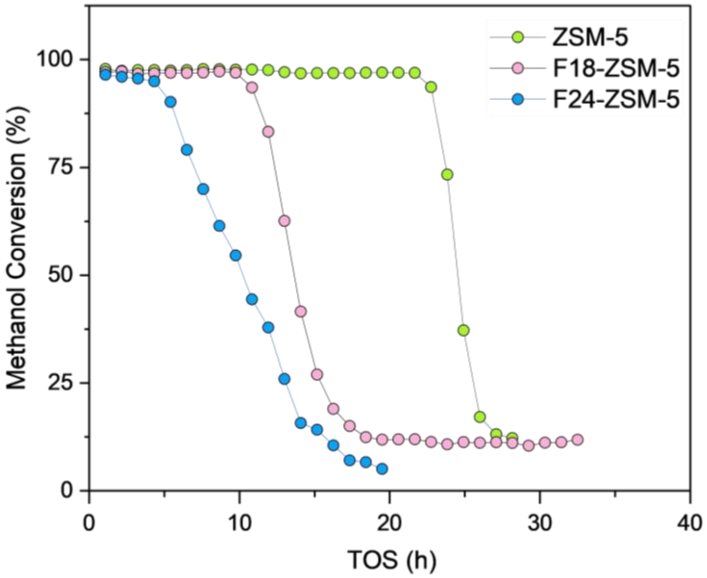

Figure S18. Catalytic performance of ZSM-5 and F-ZSM-5 (F18, and F24) catalysts at 400 °C with a WHSV of 16 h^{-1}.

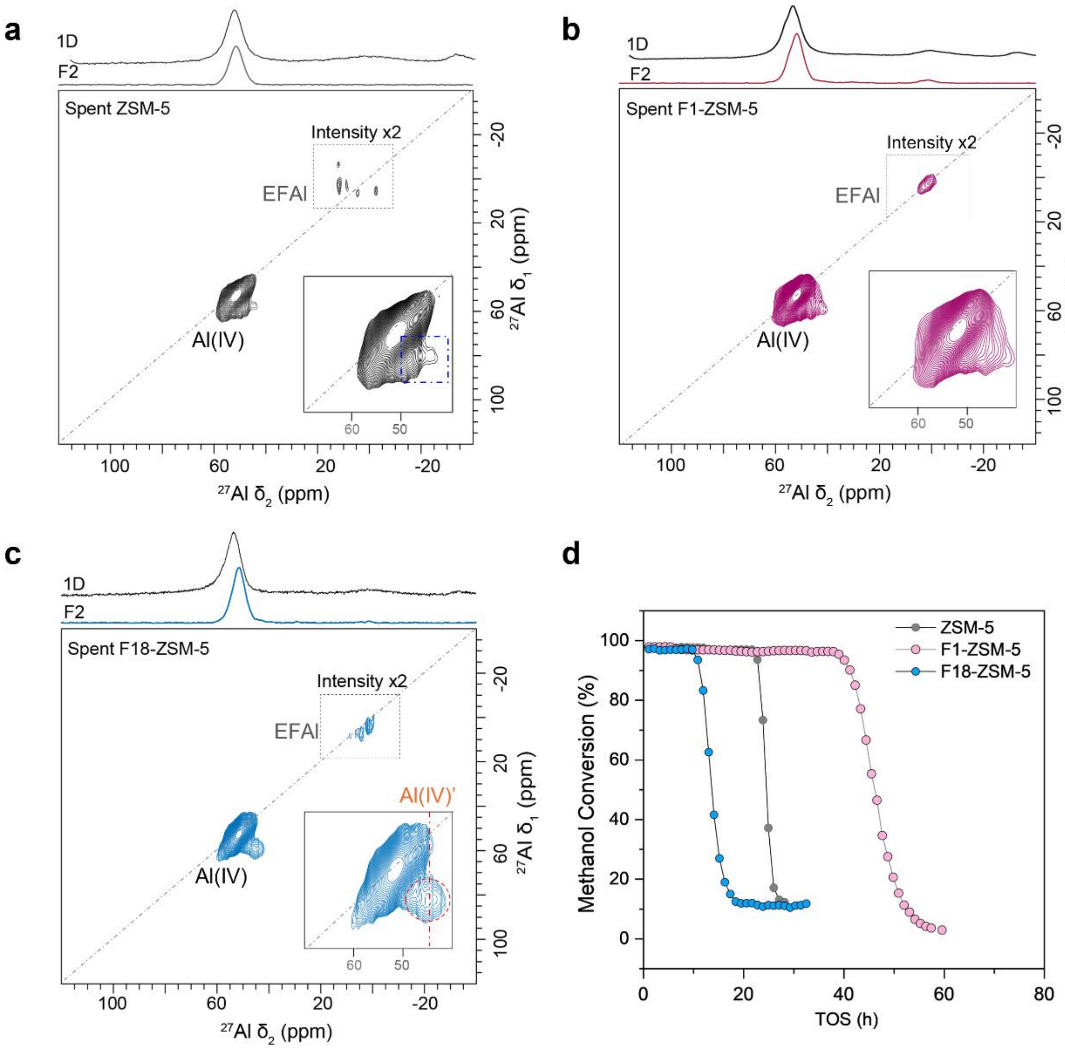

Figure S19. ^{27}Al 3QMAS NMR spectra of ZSM-5 catalysts after the MTH reaction. (a) spent ZSM-5, (b) spent F1-ZSM-5, and (c) spent F18-ZSM-5. (d) Methanol conversion of catalysts as a function of time-on-stream (TOS) for corresponding catalysts.

The 1D spectra of all samples (Figure S21a-c) are dominated by tetrahedrally coordinated framework Al, with only minor contributions from extra framework Al species (EFAl). However, distinct differences are observed in 2D ^{27}Al 3QMAS contour shapes. For the spent parent ZSM-5, a relatively well-defined Al(IV) contour centered at 53 ppm is observed, while a very weak off-diagonal signal appears at ~44 ppm in the F2 dimension (highlighting in blue rectangle, Figure S21a), while remains associated with tetrahedral Al in the isotropic dimension, suggesting a locally distorted or partially coordinated framework Al species formed during the MTH reaction, rather than to classical extra-framework Al.

In the case of F1-ZSM-5 (Figure S21b), the 2D contour becomes broadened without the appearance of distinct off-diagonal features, suggesting an increased heterogeneity and local perturbation of framework Al environments without compromising the overall framework integrity.

Notably, the F18-ZSM-5 (Figure S21c) exhibits a pronounced off-diagonal feature around ~45 ppm in the F2 dimension, suggesting the formation of strongly distorted framework Al environment after reaction.[14] This progressive increase in off-diagonal intensity and contour broadening indicates that over fluorination promotes the distortion and structural evolution, hence, leading to an extremely shorted lifetime of 9 h (Figure S21d) compare with that of parent ZSM-5 (21 h), supported by the data of XRD analysis (Figure S2) and the SEM images (Figure S5).

SAMPLE	HTI	PTO
ZSM-5	0.595	1.46
F1-ZSM-5	0.611	2.02
F3-ZSM-5	0.630	1.92
F6-ZSM-5	0.600	1.64
F18-ZSM-5	0.469	0.87

Table S2. Hydrogen transfer index (HTI, butanes/(butanes+butenes)).[15] and C_2–C_7 paraffin-to-olefin ratio (PTO) at TOS = 1 h.

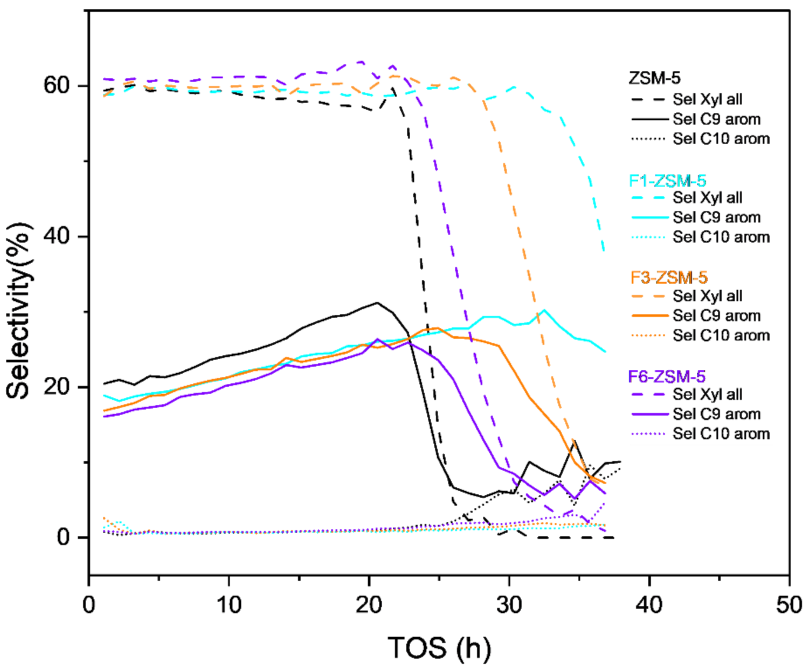

Figure S20. Selectivity of xylene, C9, and C10 aromatics in the methanol-to-hydrocarbon (MTH) reaction over parent and fluorinated ZSM-5 catalysts. The suppression of higher aromatics formation indicates that fluorination moderates the acid strength and reduces excessive secondary alkylation and cyclization reactions. This effect correlates with the NH_3-TPD and pyridine-FTIR results, which show a redistribution of acidity from strong BASs to weaker BAS-like sites. Consequently, the reduced formation of bulky polyaromatics mitigates coke deposition and extends the catalyst lifetime during the MTH reaction.

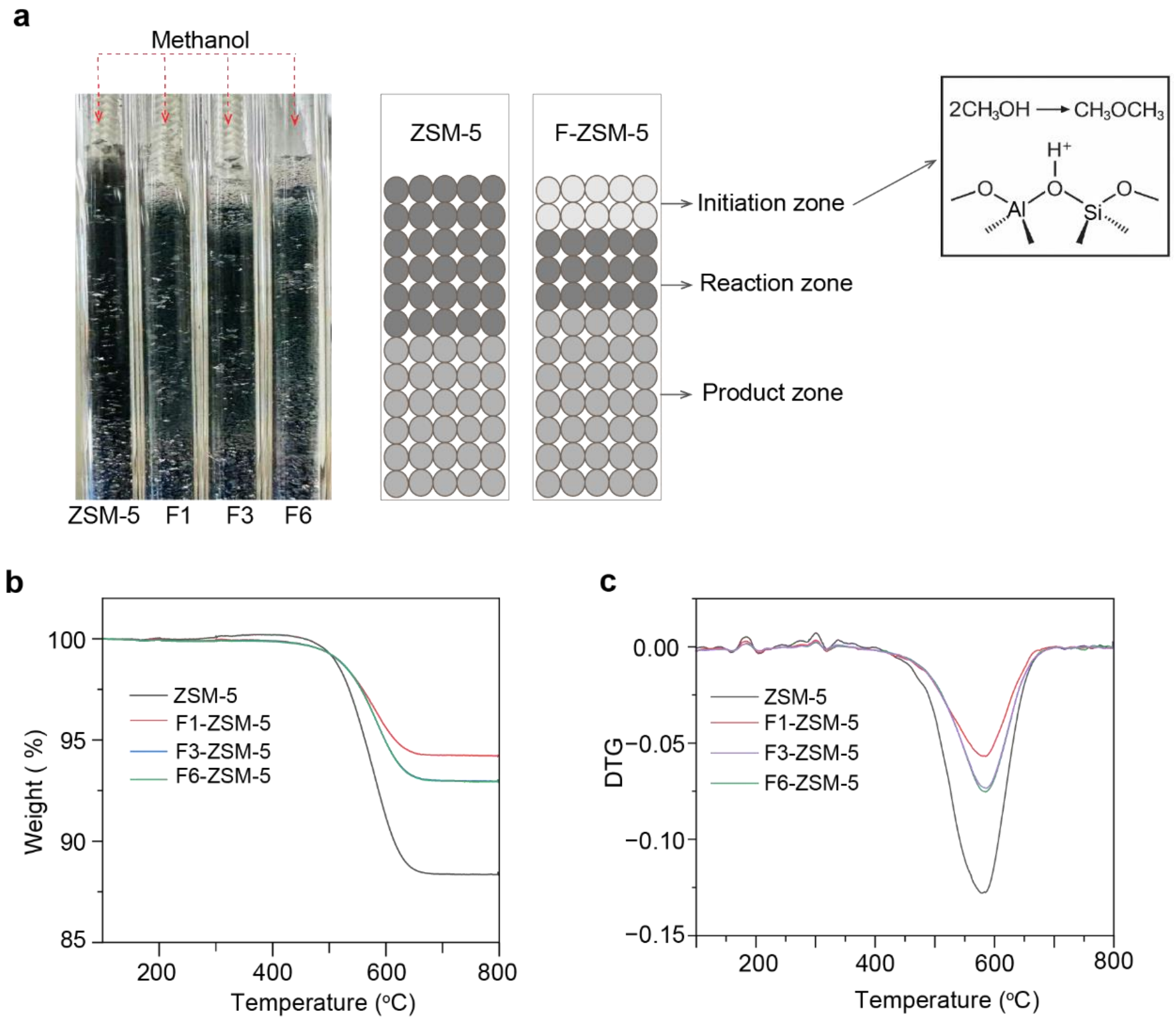

Figure S21. Analysis of spent catalysts. (a) Optical images of the reacted fixed-bed catalysts after the MTH reaction and schematic illustration of the reaction zones (initiation, reaction, and product zones) in ZSM-5 and F-ZSM-5 catalysts. (b) TGA and (c) derivative thermogravimetric (DTG) profiles of spent catalysts.

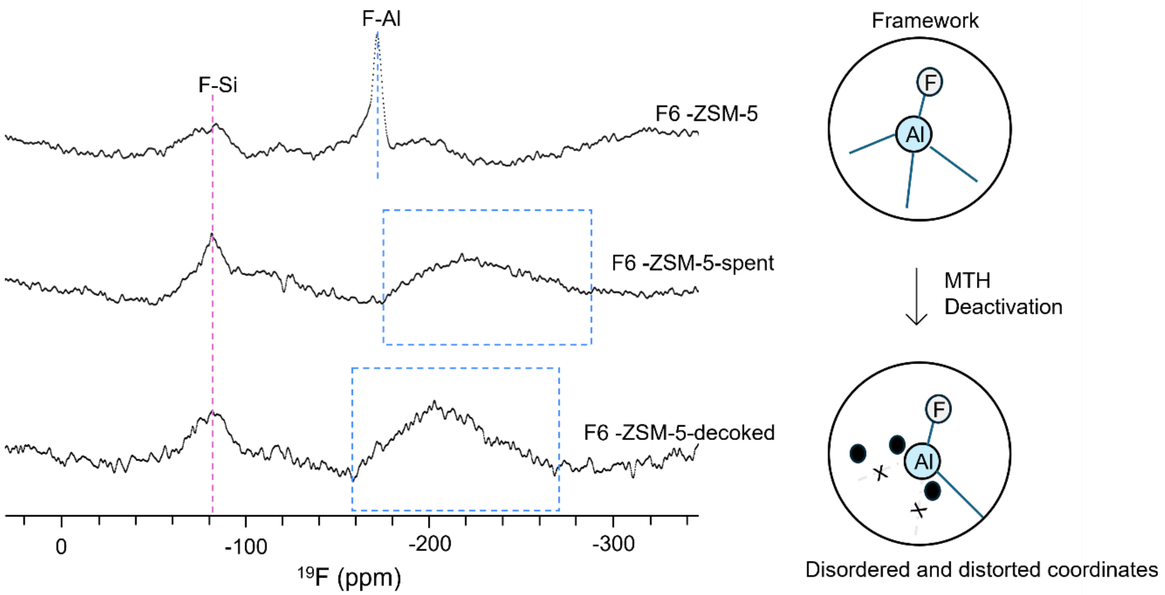

Figure S22. ^{19}F MAS NMR spectra of fresh, MTH reacted (coke-containing), and TGA-analyzed (decoked) F6-ZSM-5 catalysts.

For F6-ZSM-5 (Figure S23), the fresh catalyst exhibits well-defined resonances assigned to F-Si and F-Al species. After the MTH reaction, the F-Al resonance becomes significantly attenuated and poorly resolved, accompanied by the emergence of broad features centered near −200 ppm. This indicates that fluorine species initially coordinated to Framework Al undergo substantial structure evolution after MTH reaction, while the F-Si-related signal remains comparatively unchanged.

Notably, after thermogravimetric analysis (TGA) treatment (from 50 °C to 800 °C, air), these broad features persist without recovery of the original F-Al signal even though it turned relative narrowed compared that of F6-ZSM-5-spent. This suggests that the observed changes are not solely due to coke deposition but instead reflects irreversible structural transformation of fluorine species associated with framework Al. However, the persistent fluorine signals suggest that fluorine remains within the ZSM-5 system even after calcination at 800 °C. The observed evolution in F-Al coordination is likely attributed to the rearrangement of the framework Al environment induced during the MTH process.

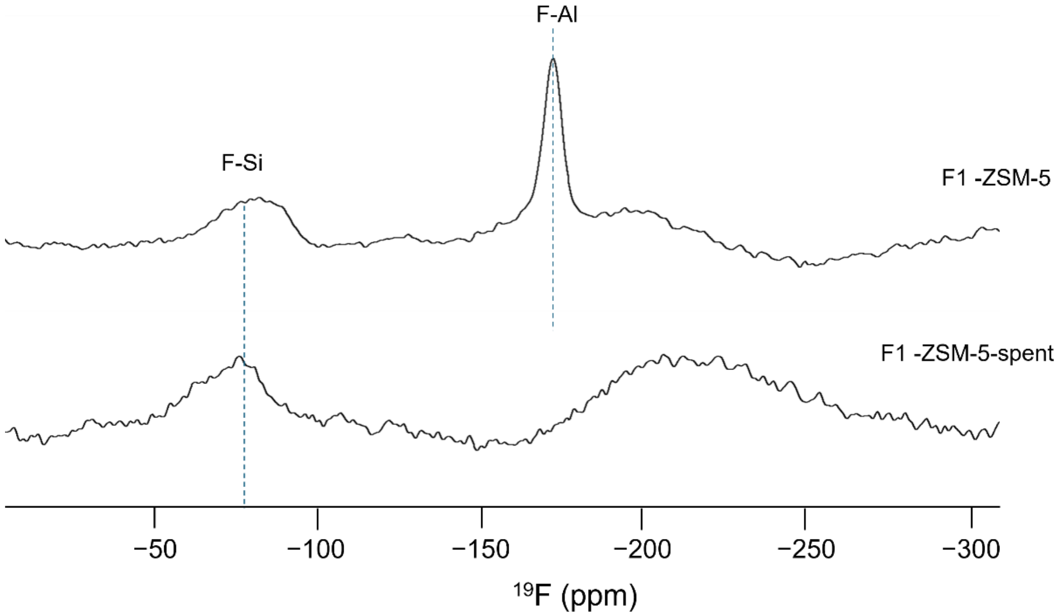

Figure S23. ^{19}F MAS NMR spectra of fresh, and MTH reacted (coke-containing) of F1-ZSM-5 catalysts. To demonstrate that the evolution of F–Al species is not limited to high fluorine loading (e.g., F6), we also examined the F1 sample. A similar trend is observed, where the F–Al coordination is significantly altered after the reaction, while the F–Si species remain relatively stable.

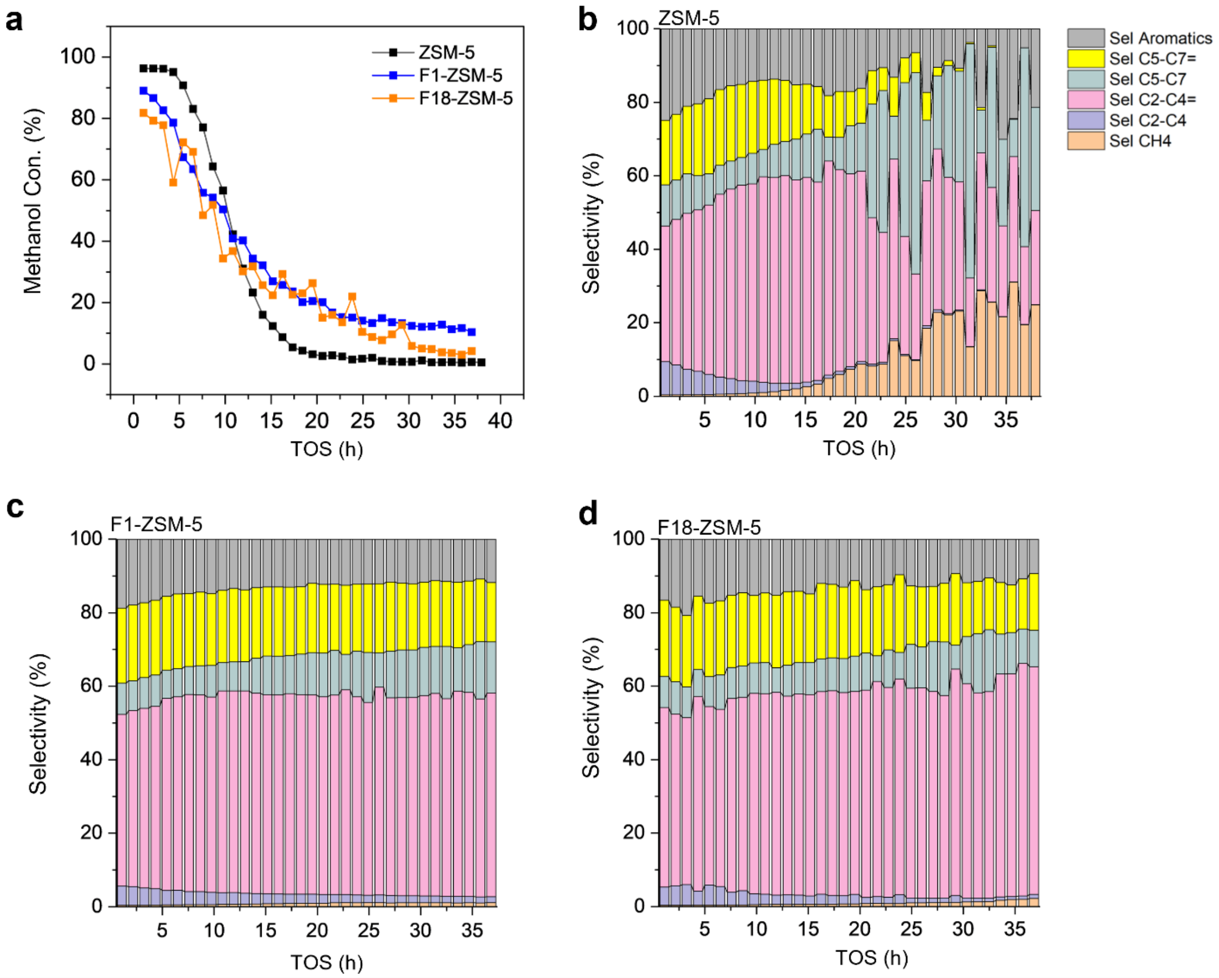

Figure S24. Catalytic performance of regenerated parent and fluorinated ZSM-5 (F1 and F18) catalysts in the MTH reaction. (a) Methanol conversion as a function of time on stream (TOS). (b–d) Product selectivity as a function of TOS: for (b) regenerated ZSM-5, (c) regenerated F1-ZSM-5, and (d) regenerated F18-ZSM-5. The catalysts were regenerated in a flowing mixture of O_2/N_2 (5/15 mL min^{-1}) at 550 °C for 6 h.

To further examine the observed transformation of F–Al species after the MTH reaction on catalytic performance and to understand the role of F-Al species in MTH better, the regenerated catalysts were re-evaluated in the MTH reaction. Compared with the regenerated parent ZSM-5 (R-ZSM-5), the regenerated F1-ZSM-5 (denoted as R-F1-ZSM-5) exhibit lower methanol conversion. This behavior consistent with the attenuation of F–Al species (–172 ppm), suggesting loss of the framework Al and intrinsic BAS acid sites.

It is worth noting that all regenerated catalysts exhibit lower aromatic selectivity compared to their fresh counterparts. For example, the aromatic selectivity of the parent ZSM-5 decreases from 30.4% in the fresh catalyst to 24.0% after regeneration. This general trend suggests that the regeneration process alters the nature of the acid environment. Such changes are likely associated with modifications of framework Al environments under reaction and may reduce the effectiveness of BASs in promoting aromatization reactions.

More importantly, the severe loss of the aromatic selectivity from 31.5% in F1-ZSM-5 to 17.2% inR-F1-ZSM-5, the regenerated F1-ZSM-5 shows even lower aromatic selectivity (17.2%) than the regenerated parent ZSM-5. Suggesting that F–Al environments play a role in enhancing hydrogen transfer and aromatization in the fresh catalyst, as further proved by the decreased HTI values in regenerated fluorinated-ZSM-5 (Table S3).

The remaining F–Si species are associated with a more stable and "cleaner" reaction behavior, characterized by suppressed methane and paraffin formation and more stable olefin production. Rather than acting as highly active acid sites, these F–Si environments are more likely to modify the local reaction environment, making it less favorable for extensive secondary reactions in the MTH process.

This behavior can also be understood from a proximity standpoint. The F–Al-associated polarized hydroxyls are likely located near BASs, enabling cooperative interactions that enhance hydrogen transfer and aromatization. In contrast, the F–Si-associated hydroxyl species are likely more spatially separated from active acid sites, reducing their ability to participate in such reactions. This is consistent with the cleaner reaction behavior observed for F–Si-dominated catalysts.

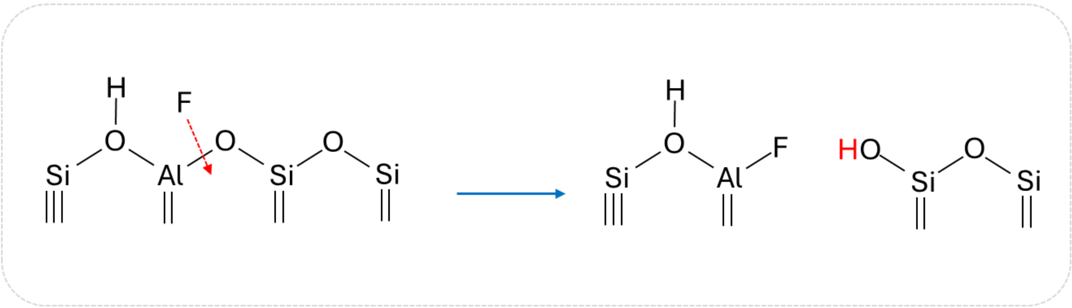

Figure S25. Schematic illustration of proposed fluorine-induced framework modification in ZSM-5. Fluorine attack on the Si–O–Al bond leads to framework cleavage, forming Al–F coordination and generating hydroxyl groups. The resulting local environments (e.g., Si–OH–Al–F) can further polarize neighboring hydroxyls, contributing to modified acidity.

Samples	HTI (Butanes/(Butanes + Butenes))
ZSM-5	0.32
F1-ZSM-5	0.25
F18-ZSM-5	0.23

Table S3. Hydrogen transfer index (HTI, butanes/(butanes+butenes)) over regenerated parent and fluorinated ZSM-5 catalysts at TOS of 1 h.

References

(1) Qin, Z.; You, Z.; Bozhilov, K. N.; Kolev, S. K.; Yang, W. Dissolution Behavior and Varied Mesoporosity of Zeolites by NH_4F Etching. *Chemistry–A European Journal,* **2022**, 266101, 1–7.

(2) Nguyen, C.; Do, D. D. The Dubinin-Radushkevich Equation and the Underlying Microscopic Adsorption Description. *Carbon N.Y.* **2001**, 39 (9), 1327–1336.

(3) Ghezini, R.; Sassi, M.; Bengueddach, A. Adsorption of Carbon Dioxide at High Pressure over H-ZSM-5 Type Zeolite . Micropore Volume Determinations by Using the Dubinin – Raduskevich Equation and the ‘‘t-Plot’’ Method. *Microporous and Mesoporous Materials* **2008**, d, 370–377.

(4) C.A. Emeis, Determination of Integrated Molar Extinction Coefficients for Infrared Absorption Bands of Pyridine Adsorbed on Solid Acid Catalysts, *Journal of catalysis,* **1993**, 141, 347-354.

(5) Wang, H.; Li, X.; Jiang, Y.; Li, M.; Xiao, Q.; Zhao, T.; Yang, S.; Qi, C.; Qiu, P.; Yang, J.; Jiang, Z.; Luo, W. A Universal Single-Atom Coating Strategy Based on Tannic Acid Chemistry for Multifunctional Heterogeneous Catalysis. *Angew. Chemie-Int.Ed.* **2022**, 61 (14), e202200465.

(6) Murali, G.; Reddeppa, M.; Seshendra Reddy, C.; Park, S.; Chandrakalavathi, T.; Kim, M. D.; In, I. Enhancing the Charge Carrier Separation and Transport via Nitrogen-Doped Graphene Quantum Dot-TiO_2 Nanoplate Hybrid Structure for an Efficient NO Gas Sensor. *ACS Appl. Mater. Interfaces* **2020**, 12 (11), 13428–13436. https://doi.org/10.1021/acsami.9b19896.

(7) Chen, H.; Gulbinski, J.; Jain, S.; Tabassum, T.; Lee, C.; Mello, D. De; Marcella, N.; Frenkel, A.; Boscoboinik, A. The Dynamic Catalytic Activity of Phosphorus-Containing Catalysts. DOI: https://doi.org/10.26434/chemrxiv-2023-g8cb5-v2

(8) Ma, J.; Ren, Y.; Zhou, X.; Liu, L.; Zhu, Y.; Cheng, X.; Xu, P.; Li, X.; Deng, Y.; Zhao, D. Pt Nanoparticles Sensitized Ordered Mesoporous WO_3 Semiconductor: Gas Sensing Performance and Mechanism Study. Adv. Funct. Mater. **2018**, 28 (6), 1–12.

(9) Brown, S. P.; Spiess, H. W. Advanced Solid-State NMR Methods for the Elucidation of Structure and Dynamics of Molecular , Macromolecular , and Supramolecular Systems. 2001.https://chemrxiv.org/doi/full/10.26434/chemrxiv-2023-g8cb5-v2

(10) Ji, Y.; Chen, K.; Han, X.; Bao, X.; Hou, G. Precise Structural and Dynamical Details in Zeolites Revealed by Coupling-Edited ^{1}H–^{17}O Double Resonance NMR Spectroscopy. **2024**.

(11) Liang, L.; Ji, Y.; Chen, K.; Gao, P.; Zhao, Z.; Hou, G. Solid-State NMR Dipolar and Chemical Shift Anisotropy Recoupling Techniques for Structural and Dynamical Studies in Biological Systems. *Chem. Rev.* **2022**, 122, 10, 9880–9942.

(12) Landripet, I.; Puškari, A. Fine Tuning of Hierarchical Zeolite Beta Acid Sites Strength. *Crystals* **2024**, *14*(1), 53**,** 1–16.

(13) Palčić, A.; Moldovan, S.; El Siblani, H.; Vicente, A.; Valtchev, V. Defect Sites in Zeolites: Origin and Healing. *Adv. Sci.* **2022**, 9 (4), 1–11.

(14) Sazama, P.; Wichterlova, B.; Dedecek, J.; Tvaruzkova, Z.; Musilova, Z.; Palumbo, L.; Sklenak, S.; Gonsiorova, O. Microporous and Mesoporous Materials FTIR and 27 Al MAS NMR Analysis of the Effect of Framework Al- and Si-Defects in Micro- and Micro-Mesoporous H-ZSM-

5 on Conversion of Methanol to Hydrocarbons. *Microporous Mesoporous Mater.* **2011**, 143 (1), 87–96.

(15) Depart-, T. E.; Gmbh, S.; Technology, M.; Engineering, B. Acidity Modification of ZSM-5 for Methane Conversion in Co-Feeding Method with MTA Reaction. *Chem. Res. Chin. Univ.* **2022**, 38 (4), 1012–1017.
